# Synaptotagmin 17 controls neurite outgrowth and synaptic physiology via distinct cellular pathways

**DOI:** 10.1038/s41467-019-11459-4

**Published:** 2019-08-06

**Authors:** David A. Ruhl, Ewa Bomba-Warczak, Emma T. Watson, Mazdak M. Bradberry, Tabitha A. Peterson, Trina Basu, Alyssa Frelka, Chantell S. Evans, Joseph S. Briguglio, Tamara Basta, Michael H. B. Stowell, Jeffrey N. Savas, Avtar Roopra, Robert A. Pearce, Robert C. Piper, Edwin R. Chapman

**Affiliations:** 1 Department of Neuroscience, University of Wisconsin, Madison, WI 53706 USA; 2 Department of Neurology, Feinberg School of Medicine, Northwestern University, Chicago, IL 60611 USA; 3 Department of Molecular Physiology and Biophysics, University of Iowa, Iowa City, IA 52242 USA; 4 Department of Anesthesiology, University of Wisconsin, Madison, WI 53706 USA; 5 Department of Physiology, Perelman School of Medicine, University of Pennsylvania, Philadelphia, PA 19104 USA; 6 Department of Molecular, Cellular and Developmental Biology, University of Colorado at Boulder, Boulder, CO 80309 USA; 7 Howard Hughes Medical Institute, Chevy Chase, MD 20815 USA

**Keywords:** Neuroscience, Cellular neuroscience, Development of the nervous system, Synaptic transmission

## Abstract

The synaptotagmin (syt) proteins have been widely studied for their role in regulating fusion of intracellular vesicles with the plasma membrane. Here we report that syt-17, an unusual isoform of unknown function, plays no role in exocytosis, and instead plays multiple roles in intracellular membrane trafficking. Syt-17 is localized to the Golgi complex in hippocampal neurons, where it coordinates import of vesicles from the endoplasmic reticulum to support neurite outgrowth and facilitate axon regrowth after injury. Further, we discovered a second pool of syt-17 on early endosomes in neurites. Loss of syt-17 disrupts endocytic trafficking, resulting in the accumulation of excess postsynaptic AMPA receptors and defective synaptic plasticity. Two distinct pools of syt-17 thus control two crucial, independent membrane trafficking pathways in neurons. Function of syt-17 appears to be one mechanism by which neurons have specialized their secretory and endosomal systems to support the demands of synaptic communication over sprawling neurite arbors.

## Abstract (web summary)

The functional role of synaptotagmin-17 (syt-17) has remained unanswered. In this study, authors demonstrate that syt-17 exists in two distinct pools in hippocampal neurons (Golgi complex and early endosomes), where it served two completely independent functions: controlling neurite outgrowth and synaptic physiology

## Introduction

Synaptotagmins (syt) are generally thought to regulate exocytosis at the plasma membrane. This protein family comprises seventeen isoforms, each possessing two C2 domains separated by a short flexible linker. The best-studied isoform, syt-1, is targeted to synaptic vesicles, where it triggers membrane fusion in response to Ca^2+^^1^. This function, regulated exocytosis of vesicular cargo, appears canonical, as structurally diverse syt isoforms appear to play similar roles. For example, syt-9 regulates FSH release from pituitary gonadotropes^2^, syt-4 regulates BDNF release in hippocampal neurons^3^, and syt-10 regulates IGF-1 release in the olfactory bulb^4^.

Despite intense interest, only a minority of syt isoforms have been assigned any specific function. A particularly neglected isoform is syt-17^5^, the most recently discovered syt, which possesses 36–44% sequence homology to syts 1–5. Unlike every other syt, syt-17 lacks an N-terminal transmembrane domain; rather, syt-17 associates with membranes via a string of fatty-acylated cysteine residues near the N-terminus of the protein^6^.

Conflicting data exist regarding the distribution of syt-17, although all reports find high levels of expression in hippocampus, particularly in pyramidal cell layers^5–8^ and in cultured hippocampal neurons^9^. Although syt-17 mRNA is also detectible in kidney (hence the original name, “B/K protein”^5^), syt-17 protein expression appears restricted to brain^6^. Similarly, inconsistent data exists regarding the subcellular localization of the protein^6,7,10,11^. While the function of syt-17 has not been determined, expression of the protein is known to increase following kainite-induced seizures^10^ or transient ischemia^12–14^. Hence, expression of syt-17 is dynamically regulated.

In the present work, we localized syt-17 in primary hippocampal neurons to two compartments: the Golgi complex in the soma and Rab5-positive early endosomes in neurites. We conducted the first characterization of a newly generated syt-17 KO mouse and found that deletion of this protein results in impaired neurite outgrowth. Further experiments indicated that the outgrowth defect is due to a disruption of the early secretory pathway. Syt-17 interacts with resident Golgi proteins to control import of cargo from the ER. This regulation of neurite outgrowth is bidirectional, as overexpression of syt-17 increases axonal length and accelerates axonal regrowth following injury. Surprisingly, neurons lacking syt-17 show a substantial increase in glutamatergic synaptic transmission, attributable to a pathological accumulation of AMPA-type glutamate receptors on the postsynaptic membrane (apparently the consequence of an observed endocytic defect), and impaired synaptic plasticity. We conclude that syt-17 is a multifunctional regulator of intracellular protein trafficking in excitatory hippocampal neurons, modulating both neural development and synaptic physiology.

## Results

### Unusual biochemical properties and localization of syt-17

**Fig. 1:**
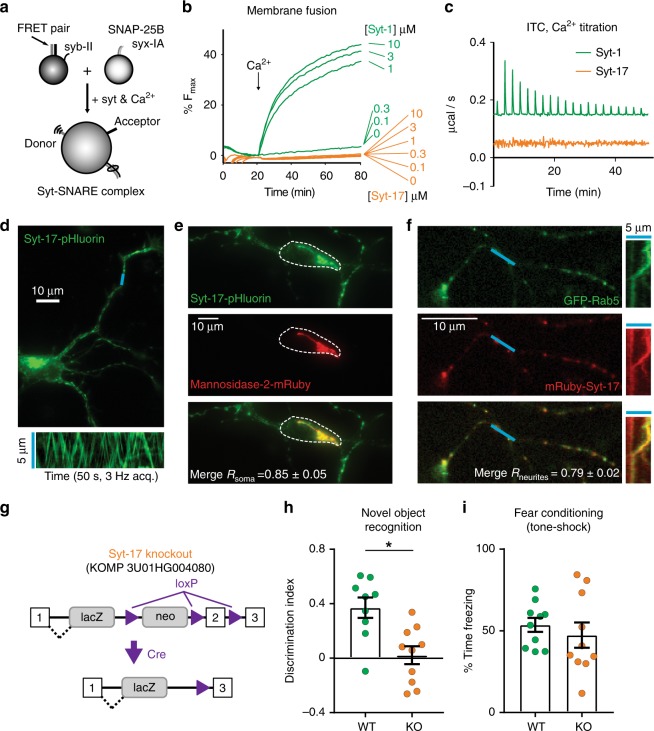
Syt-17 does not trigger membrane fusion or bind Ca^2+^, localizes to the Golgi and early endosomes, and knockouts exhibit hippocampal-dependent memory deficits. **a** Schematic of the fusion assay. **b** Syt-1 (green) and syt-17 (orange) protein titration in the fusion assay. “C2AB” indicates that the tandem C2 domains of syt-1 or syt-17 were used for experiments. Syt-17, in contrast to syt-1, is unable to stimulate fusion at all protein concentrations tested. **c** Heat of Ca^2+^ binding to isolated C2AB domains of syt-1 and syt-17 as measured by isothermal titration calorimetry. Note the lack of appreciable binding by syt-17. **d** Top: 3 DIV hippocampal neuron expressing syt-17-pHluorin. Scale bar indicates 10 μm. Bottom: Kymograph of linescan indicated in blue. Syt-17-pHluorin puncta are highly mobile and traffic bidirectionally. **e** Syt-17-pHluorin coexpressed with the Golgi marker mannosidase-2-mRuby. Colocalization (Pearson’s): 0.85 ± 0.05. Soma indicated with a dashed line. **f** Left: mRuby-syt-17 coexpressed with GFP-Rab5. Colocalization (Pearson’s): 0.79 ± 0.02. Right: Kymograph of indicated linescan, showing co-trafficking of syt-17/Rab5. Measurements from five independent preparations, 3–8 FOVs/prep. **g** Schematic of the genetic strategy. Intron 2 and the floxed Neo cassette were excised by performing a cross with a germline-expressing Cre mouse, resulting in nonsense-mediated degradation of the transcript. **h** Six-week-old mice of either gender, from > 3 breeding lines, were subject to a novel object recognition task, a test of hippocampal-dependent memory. Syt-17 KO showed significantly impaired performance relative to WT (*t*_17_ = 3.518, *p* = 0.003, *r*^2^ = 0.421, Discrimination index mean_wt_ = 0.37 ± 0.08, mean_ko_ = 0.02 ± 0.06, *N*_wt_ = 9 and *N*_ko_ = 10 animals). Syt-17 KO data points are depicted in orange and WT in green in all figures. **i** No difference was observed between WT and KO mice in hippocampal-independent paired tone-shock fear conditioning (*p* > 0.1, two-sample *t*-test). Error bars indicate S.E.M.s

Given that many syt isoforms trigger SNARE (soluble N-ethyl maleimide-sensitive factor attachment protein receptor)-catalyzed membrane fusion, we functionally compared the canonical isoform, syt-1, with syt-17 (domain structures in Supplementary Fig. 1A) with a FRET-based in vitro fusion assay^15^ (Fig. 1a). Unlike those of syt-1, the C2 domains of syt-17 had no effect on SNARE-mediated fusion (Fig. 1b). Further, unlike syt-1, syt-17 showed no apparent binding to Ca^2+^ (Fig. 1c) or phospholipids (Supplementary Fig. 1B-C). Syt-17 is thus a quite unusual syt isoform, lacking both a transmembrane domain and the biochemical properties of canonical syts.

To examine the localization of syt-17 in developing hippocampal neurons, we tagged it with superecliptic pHluorin and overexpressed it at 3 days in vitro (DIV; Fig. 1d). Two pools of syt-17 were immediately apparent: a large immobile pool in the soma, and a pool of smaller puncta that underwent antero- and retrograde transport in neurites (Fig. 1d, bottom). The localization of the immobile somatic pool of syt-17 resembled the Golgi complex, so we performed two-color imaging using syt-17-pHluorin and the Golgi marker mRuby-mannosidase-II and found the two colocalized (Fig. 1e). The behavior of the mobile syt-17 puncta subjectively resembled endosomes. We therefore co-expressed syt-17-mRuby with Rab5-GFP (Fig. 1f). Virtually all of the small syt-17 puncta in neurites were colocalized, and cotrafficked, with Rab5-GFP (Fig. 1f, right). We have found that overexpression of syt isoforms can in certain conditions induce spurious localization results, as some syts can spillover to non-physiological compartments when strongly overexpressed (unpublished observations, also see ref. ^11^). Such spillover may account for some of the previous discrepancies regarding syt-17 localization^6,7,10,11^. We therefore performed localization experiments in parallel using syt-17 that had been expressed at very low levels (with low-titer lentivirus instead of sparse transfection) and detected with a HaloTag/ligands. The same pattern of localization was observed. In summary, syt-17 localizes to two compartments in hippocampal neurons: the Golgi and early endosomes.

### Syt-17 regulates neurite outgrowth and regeneration

We obtained (Fig. 1g) and validated (Supplementary Fig. 2A) an uncharacterized syt-17 knockout (KO) mouse line. The mice exhibit significantly impaired memory function in a novel object recognition task (Fig. 1h). This deficit appears specific to hippocampal-dependent memory, as no difference between genotypes was observed in hippocampal-independent fear conditioning (Fig. 1i). No other behavioral (Supplementary Fig. 2B-D) or reproductive abnormalities were noted.

**Fig. 2:**
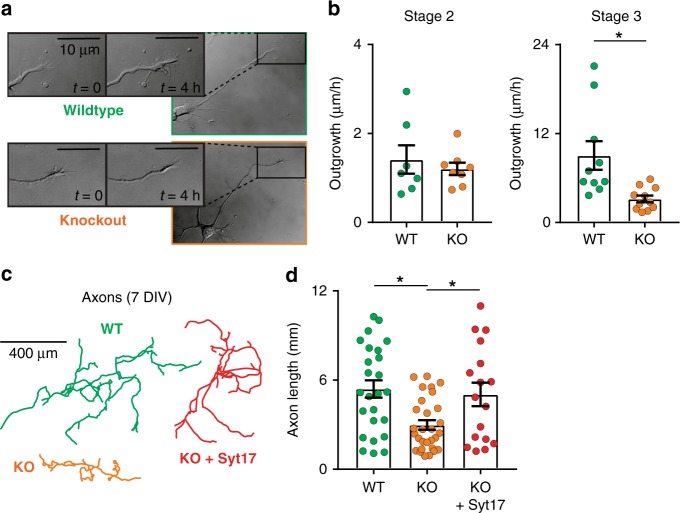
Syt-17 mediates axon outgrowth. **a** Time-lapse DIC images of stage 3 neurons from wildtype (WT, top) and syt-17 knockout (KO, bottom) animals. Insets: growth cones at higher zoom. Scale bar indicates 10 μm. **b** Neurite outgrowth is normal at stage 2 (left, *p* > 0.1) but slowed at stage 3 (right, *t*_19_ = 3.095, *p* = 0.006, *r*^2^ = 0.335, mean_wt_ = 9.04 ± 1.93 μm/h, mean_ko_ = 3.18 ± 0.46, *N*_wt_ = 10 and *N*_ko_ = 11 neurons) in KOs. **c** Axonal morphology at 7 DIV. **d** Axon length was significantly reduced in syt-17 KO neurons (*F*_2,68_ = 6.65, *p* = 0.002, post-hoc of WT vs. KO: *t*_41_ = 4.397, *p* < 0.001, *r*^2^ = 0.32, mean_wt_ = 5.23 ± 0.68 mm, mean_ko_ = 3.15 ± 0.31, *N*_wt_ = 19 and *N*_ko_ = 24 neurons), and was rescued by exogenous expression of syt-17 (red; post-hoc of rescue vs. WT: *p* < 0.1, mean_rescue_ = 5.03 ± 0.79, *N*_rescue_ = 17 neurons). Experiments were performed on 3–4 independent preparations of animals. Error bars indicate S.E.M.s

During the course of overexpression experiments, we anecdotally observed that upregulation of syt-17 in hippocampal neurons seemed to affect neurite length and morphology. Long-term imaging of developing neurons (Fig. 2a) revealed that, once polarized, stage three^16^ KO neurons extended axons approximately half as fast as WT neurons (Fig. 2b; stage two, left; stage three, right). Further, while KO neurons exhibited a similar number of primary neurites (Supplementary Fig. 3A), their growth cones spontaneously collapsed more often (Supplementary Fig. 3B) and were smaller, lacking prominent lammelipodia (Supplementary Fig. 3C-D). In principle, these effects could be due to a cytoskeletal perturbation, however no differences in the rate of retrograde actin flow^17^ or other cytoskeletal abnormalities were observed (Supplementary Fig. 3E-F). Correspondingly, more mature cells exhibit shorter neurons at 7 DIV (Fig. 2c, d), and exogenous expression of syt-17 in KO neurons rescued this deficit (Fig. 2d, right).

**Fig. 3:**
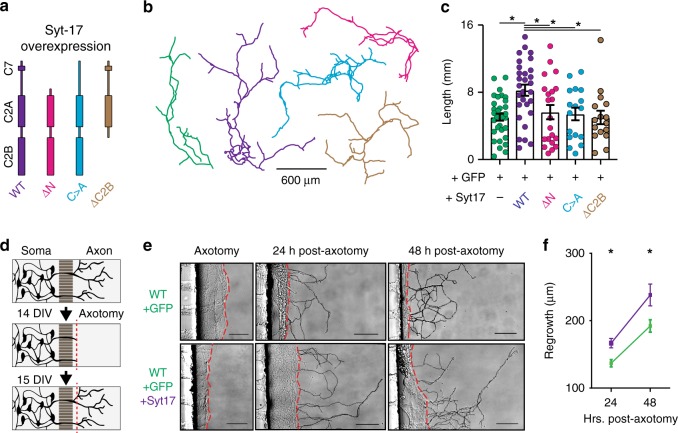
Syt-17 overexpression increases axonal length and facilitates regrowth after injury. **a** Overexpression in WT neurons of WT syt-17 (purple), and mutants either lacking the N-terminal domain (magenta), with the N-terminal cysteine patch mutated to alanines (blue), or lacking the C2B domain (brown), relative to control neurons (green). **b** Measurement of axonal length in neurons overexpressing our syt-17 constructs, or neurons expressing only GFP (green). Measurements were performed identically to Fig. 2. **c** Overexpression of syt-17 increased axon length (*F*_4,111_ = 4.79, *p* = 0.001, post-hoc of overexpression vs. control: *t*_63_ = 3.89, *p* = 0.0007, *r*^2^ = 0.193, mean_wt_ = 5.05 ± 0.42 mm, mean_overexp_ = 8.25 ± 0.67, mean_delta-N_ = 5.64 ± 0.85, mean_C>A_ = 5.42 ± 0.75, mean_delta-C2B_ = 5.01 ± 0.8, *N*_wt_ = 33, *N*_overexp_ = 29, *N*_delta-N_ = 21, *N*_C>A_ = 17, *N*_delta-C2B_ = 16), and this effect was abolished in either of the N-terminal mutants or the C2B deletion (post-hoc *t*-tests vs. control *p* > 0.1). **d** Diagram of a microfluidic culture device, illustrating axons traversing the microchannels. Following mechanical axotomy, processes re-extend into the axon chamber. **e** Representative DIC images of microfluidics with neurons virally expressing control or syt-17 lentivirus after axotomy. Regrowing axons are traced in black. Scale bar indicates 50 µm. **f** Axons are significantly longer in cells overexpressing syt-17 at both 24 (t_608_ = 3.084, *p* = 0.002, *r*^2^ = 0.015, mean_wt_ = 139.85 ± 5.27 μm, mean_overexp_ = 164.83 ± 6.05, *N*_wt_ = 290 and *N*_overexp_ = 320) and 48 (*t*_290_ = 2.632, *p* = 0.008, *r*^2^ = 0.023, mean_wt_ = 192.33 ± 9.24, mean_overexp_ = 238.3 ± 16.27, *N*_wt_ = 174 and *N*_overexp_ = 118) hours after axotomy. All error bars indicate S.E.M.s

Conversely, we found that syt-17 overexpression increased axonal length (by ~40%, Fig. 3a–c). As mentioned above, syt-17 associates with membranes via a patch of seven fatty-acylated cysteine residues near it’s N-terminus^6^. To determine if membrane localization of the protein is necessary for this phenotype, we overexpressed syt-17 mutants in which either the N-terminal region had been deleted or these cysteines had been mutated to alanines (Fig. 3a–c and Supplementary Fig. 4A, magenta and blue, respectively). Either of these mutations abolished the overexpression phenotype (Fig. 3c), showing that the ability of syt-17 to drive axonal growth critically depends on this N-terminal cysteine-rich patch. We also measured outgrowth in neurons expressing a mutant in which the C2B domain had been deleted, for reasons that are detailed below; this mutant similarly failed to produce the overexpression phenotype, showing that syt-17 requires both the C2B domain and the N-terminal cysteine cluster to drive axonal outgrowth.

It occurred to us that the accelerated axonal growth in neurons overexpressing syt-17 could one day provide a useful approach for augmenting of axonal regeneration following injury. To explore this idea, neurons were cultured in microfluidic devices in which axons of neurons seeded in one chamber traverse long (450 µm) channels to an opposing (axon-only) chamber (Fig. 3d). Isolated axons can then be mechanically severed to observe regrowth (Fig. 3d, middle and bottom). Neurons overexpressing syt-17 regrew significantly faster than control neurons expressing only GFP (Fig. 3e, f). Conversely, severed KO neurons exhibited slowed, but not abolished, regrowth relative to WT neurons (Supplementary Fig. 4B-C). Syt-17 should thus be considered a potential candidate for therapeutic interventions after axonal injury^18^.

**Fig. 4:**
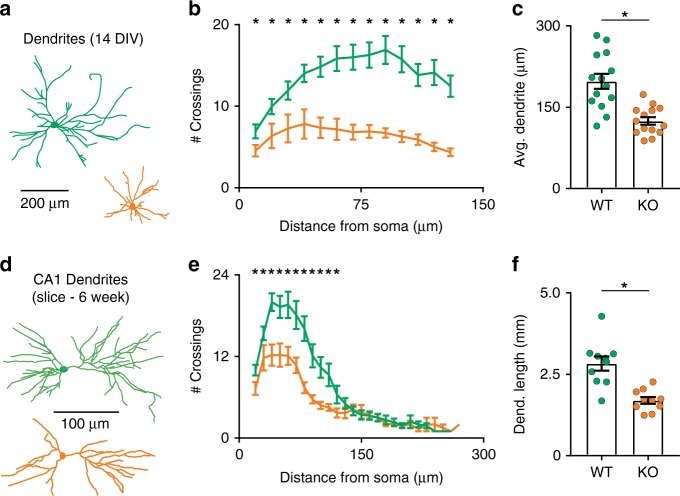
Stunted dendritic morphology in syt-17 KO neurons in vitro and in vivo. **a** Neuron morphology at 14 DIV was visualized with sparse transfection of GFP, and dendritic arbors were semi-manually reconstructed based on the presence of dendritic spines and positive MAP-2 immunostaining. **b** Sholl analysis demonstrating less dendritic branching complexity in neurons lacking syt-17. **c** The average primary dendrite length was significantly shorter in syt-17 KO neurons (*t*_26_ = 4.731, *p* < 0.001, *r*^2^ = 0.463, mean_wt_ = 197.86 ± 13.8 μm, mean_ko_ = 124.8 ± 6.93, *N*_wt_ = 14 and *N*_ko_ = 14 neurons). **d** Dendritic morphology of hippocampal CA1 pyramidal neurons was assessed with Golgi staining, and dendritic arbors from resultant slices were semi-manually reconstructed (two dimensional projections of three dimensional reconstructions are shown). **e** Sholl analysis indicated less complex dendritic branching in neurons lacking syt-17 in vivo. **f** Total dendritic length was significantly reduced in neurons lacking syt-17 (*t*_18_ = 4.653, *p* < 0.001, *r*^2^ = 0.382, mean_wt_ = 2.83 ± 0.22 mm, mean_ko_ = 1.7 ± 0.1, *N*_wt_ = 10 and *N*_ko_ = 10 neurons). Experiments were performed on 3–4 independent preparations of animals. All error bars indicate S.E.M.s

Finally, we sought to determine if these effects on neurite outgrowth are specific to axons. We examined the dendritic arbors of mature (14 DIV) neurons, and observed that those of KO neurons were shorter and less complex (by Sholl analysis, Fig. 4a–c). To validate this stunted neurite phenotype in vivo, brain sections from six-week-old postnatal mice were Golgi stained, and pyramidal neurons from hippocampal CA1 were reconstructed (Fig. 4d). These experiments revealed a defect in neurite arborization in vivo similar to that observed in cultured cells (Fig. 4e, f). Interestingly, while syt-17 KOs exhibit stunting of both axons and dendrites, the overexpression phenotype is specific to axons (Supplementary Fig. 4D-E). These results indicate that syt-17 KO neurons, in vitro and in vivo, exhibit stunted neurites due to deficient outgrowth.

### Syt-17 controls trafficking in the early secretory pathway

**Fig. 5:**
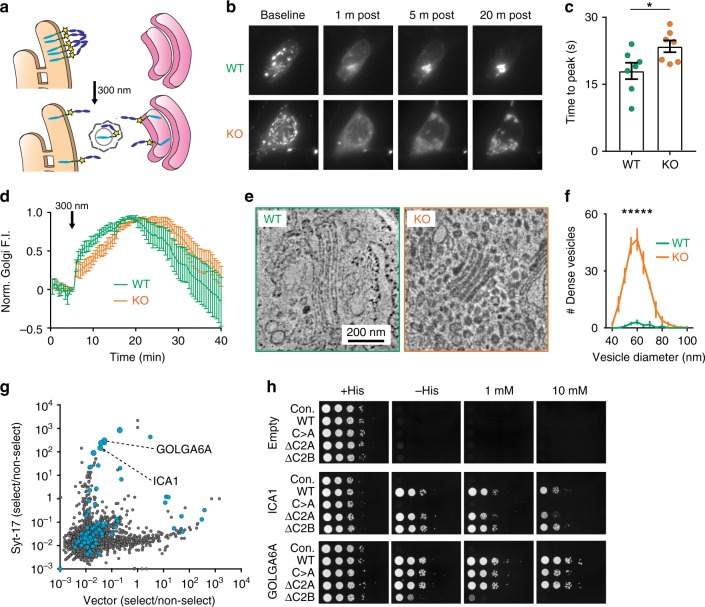
Syt-17 regulates the early secretory pathway and interacts with Golgi resident proteins. **a** VSVG-YFP-2xUVR8 aggregates in the ER in the dark (top), disaggregates upon illumination at 300 nm, and is trafficked to the Golgi (bottom). **b** Representative images of uncaging and accumulation in WT and KO neurons. **c** Syt-17 KOs exhibit a slower time to peak Golgi fluorescence (*t*_12_ = 2.425, *p* = 0.03, *r*^2^ = 0.329, mean_wt_ = 18 ± 1.84 s, mean_ko_ = 23 ± 1.33, *N*_wt_ = 7 and *N*_ko_ = 7 neurons). **d** Time course for each cell. In KO neurons (orange) cargo accumulates more slowly. Measurements made from three independent preparations. All error bars indicate S.E.M.s. **e** Representative electron micrograph of the somatic Golgi complex in WT (left) and KO (right) neurons, demonstrating vesicle accumulation in the KO. Scale bar indicates 200 nm. **f** Histogram of vesicle diameters quantified across four fields of view from two litters of mice. Error bars indicate S.E.M.s. **g** Result of a DEEPN analysis for syt-17 interactors. Plotted is the ratio of each gene abundance in the selected (-His grown) sub-population vs. the non-selected (+His) subpopulation (see Methods section). Genes for which the majority of plasmids were in the proper reading frame with respect to the Gal4 activation domain (i.e., potential interactors) are shown in blue. Two Golgi proteins among the top hits, GOLGA6A and ICA1, are indicated. **h** Binary yeast two-hybrid assays showing interaction of full-length (WT) syt-17, and the indicated syt-17 mutants, with Golgi proteins GOLGA6A and ICA1. Incubations with 1 mM and 10 mM of 3-aminitriazole (see Methods) are shown on right

The fact that much of syt-17 localizes to the Golgi prompted us to wonder if a role in the secretory pathway could explain the regulation of neurite morphology. Beginning at the earliest step of Golgi membrane transport, we assayed for alterations in trafficking of an exogenously expressed cargo^19,20^ from the ER to the early Golgi (Fig. 5a). In neurons lacking syt-17, the probe accumulated more slowly in the Golgi following uncaging (Fig. 5b–d), revealing that the kinetics of membrane trafficking in the early secretory pathway are slowed in the KO. At the ultrastructural level, we observed that a large number of darkly-stained vesicles had accumulated around the Golgi complex in syt-17 KO neurons, but not in WT (Fig. 5e). The size distribution (~60 nm average diameter, Fig. 5f) and density of these organelles is consistent with ER-to-Golgi transport vesicles^21^. Further, proteomic analysis of hippocampal lysates from six-week-old mice revealed widespread alterations in vesicular and membrane proteins in the KO, including a significant upregulation of Sec23a (Supplementary Fig. 5A-C and Supplementary Data 1), a core element of the COP-II coat present on ER-to-Golgi trafficking vesicles. The retardation of ER-to-Golgi trafficking observed in Fig. 5b–d thus appears to result from an inability of post-ER vesicles to fuse with the Golgi complex, which instead accumulate around the Golgi in the KO.

Impaired ER-to-Golgi trafficking is sufficient to stunt neurite development^22,23^, and produces a phenotype of accumulated peri-Golgi vesicles similar to what we have observed here^22,24^. The defect in early secretory trafficking thus plausibly accounts for the observed stunting of neurite outgrowth in syt-17 KO neurons.

It seemed possible to us that syt-17 may be playing a role in the Golgi analogous to that syt-1 plays in synapses: triggering SNARE-catalyzed vesicular fusion. However, syt-17 played no stimulatory role for membrane fusion mediated by presynaptic SNAREs in vitro (Fig. 1b), and pull-down assays probing for interactions between syt-17-Halo with more relevant SNAREs yielded null results (Supplementary Fig. 6A). To search more systematically for potential interactors, we conducted a yeast two-hybrid analysis (Supplementary Fig. 6B) and found that a golgin, GOLGA6A, was the highest-rated potential interactor (Fig. 5g and Supplementary Data 2). The cis-Golgi protein GOLGA6A is not well-characterized, but has the highest sequence homology (~50%) with another cis-Golgi golgin, GM130, known to play a role in the tethering and import of vesicles from the ER^25,26^. Interestingly, among the top hits was another Golgi protein, ICA1 (Fig. 5g and Supplementary Data 2), which interacts with Rab2 to regulate trafficking of vesicles from the ER to the Golgi^27^. We mapped the syt-17-interacting domains to specific structural elements of these two proteins: to the coiled-coil domain of GOLGA6A, and a region encompassing the arfaptin homology domain (AHD) of ICA1 (Supplementary Fig. 6C). We validated these two candidates in binary yeast two-hybrid assays (Fig. 5h, Supplementary Fig. 6D-E), using various deletions or mutations of syt-17 as bait. These experiments confirmed that these two Golgi proteins, implicated in import of cargo from the ER, are bona fide syt-17 interactors. The interaction with GOLGA6A was disrupted by deleting the C2B domain of syt-17 (Fig. 5h, bottom row), while the interaction with ICA1 was abolished when the N-terminal cysteines of syt-17 were substituted with alanine residues (Fig. 5h, middle row). We reiterate that both of these mutant forms of syt17 failed to enhance neurite outgrowth (Fig. 3a–c). These experiments show that two distinct domains of syt-17 physically interact with two key Golgi proteins implicated in the tethering and import of cargo from the ER. Further, syt-17 must interact with these two proteins to drive axonal outgrowth, consistent with the idea that action of syt-17 at the Golgi complex is requisite for normal neurite development.

In sum, we find that syt-17 regulates early secretory trafficking by forming a complex with resident Golgi proteins to mediate efficient import of cargo.

### Endosomal syt-17 regulates postsynaptic receptor function

**Fig. 6:**
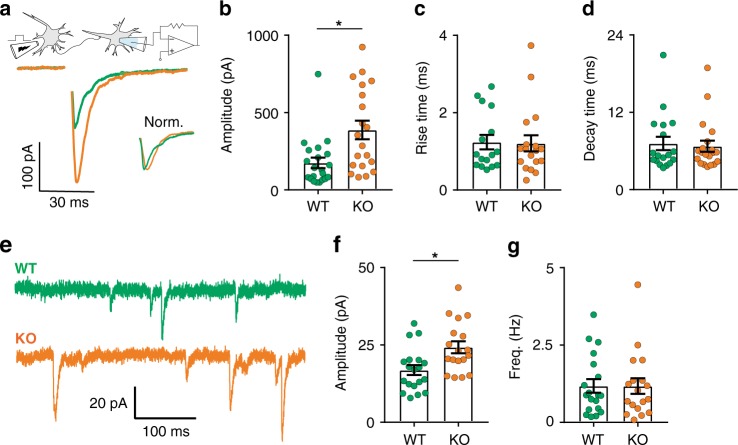
Syt-17 KO neurons exhibit enhanced synaptic responses. **a** Average AMPAR-EPSC waveforms from WT and KO neurons; amplitude-normalized traces shown in inset. **b** Syt-17 KO neurons exhibit significantly higher EPSC amplitudes (*t*_39_ = 3.118, *p* = 0.003, *r*^2^ = 0.2, mean_wt_ = 175.2 ± 34.47 pA, mean_ko_ = 387.96 ± 59.84, *N*_wt_ = 21 and *N*_ko_ = 20 neurons). Rise (**c**) and decay (**d**) times did not differ (*p* > 0.1, two-sample *t*-test). **e** Whole-cell patch clamp recordings were made from 14–17 DIV hippocampal neurons in the presence of TTX to block action potentials and AP-V and picrotoxin to isolate AMPAR currents. **f** The average amplitude of spontaneous mEPSCs was significantly larger in syt-17 KO neurons (*t*_35_ = 2.951, *p* = 0.006, *r*^2^ = 0.199, mean_wt_ = 16.85 ± 1.6 pA, mean_ko_ = 24.24 ± 1.94, *N*_wt_ = 19 and *N*_ko_ = 18 neurons). **g** No alteration was detected in presynaptic release frequency (*p* > 0.1 two-sample *t*-test). Experiments were performed on 3–4 independent preparations of animals. All error bars indicate S.E.M.s

**Fig. 7:**
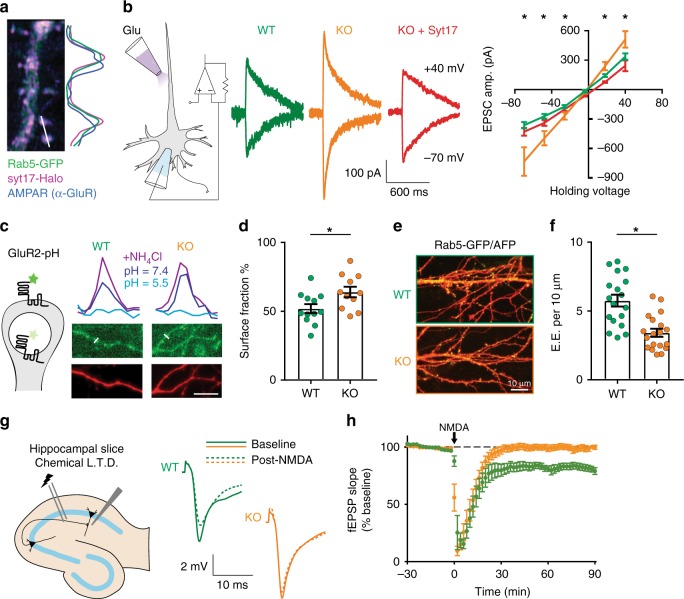
Alterations in endocytic recycling associated with accumulation of postsynaptic AMPA receptors and defective synaptic plasticity, in syt-17 KOs. **a** Colocalization of syt17 (Halo fusion construct, magenta), early endosomes (Rab5-GFP, green), and AMPA receptors (anti-GluR, blue) in dendritic spines of a 14 DIV hippocampal neuron. The traces to the right represent normalized fluorescence from the indicated 3 µm linescan. **b** Left: L-Glu was pressure-applied to apical dendrites. Middle: Representative traces of AMPAR Glu response at −70 and +40 mV in WT, KO, and KO+rescue neurons. Right: I–V plot showing current as a function of holding voltage. The amplitude of postsynaptic responses in syt-17 KO neurons was uniformly increased (except near the reversal potential), and this effect was rescued by expression of exogenous syt-17. **c** Left: GluR2-pHluorin was exogenously expressed in WT and KO neurons. Right: Spine fluorescence of GluR2-pHluorin was quantified in ACSF of pH 7.4 (extracellular pH) and 5.5 (vesicular pH), and in the presence of NH_4_Cl to unquench all pHluorin. Traces show intensity at indicated linescans. **d** Surface expression of GluR2-pHluorin was increased in syt-17 KO (*t*_21_ = 2.417, *p* = 0.02, *r*^2^ = 0.218, mean_wt_ = 51.95 ± 3.27% surface fraction, mean_ko_ = 64.01 ± 3.81, *N*_wt_ = 12 and *N*_ko_ = 11 neurons). **e** Rab5-GFP was expressed in WT and KO neurons. **f** KO neurons had significantly fewer early endosomes per unit dendrite (*t*_35_ = 4.529, *p* < 0.001, *r*^2^ = 0.37, mean_wt_ = 5.75 ± 0.43 early endosomes per 10 μm dendrite, mean_ko_ = 3.42 ± 0.29, *N*_wt_ = 18 and *N*_ko_ = 19 neurons). Scale bar indicates 10 μm. All experiments were performed on 3–4 independent preparations of animals. **g** Chemical long-term depression (LTD) along the Shaffer collaterals in hippocampal slices. Right: Representative field excitatory postsynaptic potentials (fEPSPs, 50% of maximal) before (solid lines) and after (dashed lines) five-minute NMDA application. **h** KO slices fail to exhibit LTD following the induction protocol (*p* = .012 two-sample *t*-test post-induction). Experiments were performed on slices from five animals per genotype. All error bars indicate S.E.M.s

Based on the secretory pathway defect reported above, we hypothesized that syt-17 KO neurons would exhibit a corresponding reduction in synaptic transmission, as has been observed in other models in which secretory trafficking has been disrupted^22,28^. On the contrary, we observed a dramatic and unexpected increase in the strength of excitatory responses (Fig. 6a, b), without any change in kinetics (Fig. 6c, d). This effect could not be explained by an increase in synaptic density (Supplementary Fig. 7A-B) or in presynaptic Ca^2+^ influx (Supplementary Fig. 7C-D). We did, however, detect an increase in the amplitude of miniature release events (Fig. 6e–g), potentially implicating a postsynaptic alteration. Indeed, we noted that syt-17 colocalized with both Rab5 and AMPA receptors in neuronal dendrites (Fig. 7a). To bypass the presynapse entirely, brief pulses of glutamate were applied directly to apical dendrites at consistent locations 60 µm from somata (Fig. 7b, left). We observed a significant increase in the postsynaptic responsiveness of syt-17 KO neurons that could be rescued by exogenous expression of syt-17 (Fig. 7b). Importantly, this increase fully accounted for the increase observed with presynaptic stimulation (an approximate doubling in both cases). We further observed an increase in the number of AMPA-type glutamate receptors on the postsynaptic surface of syt-17 KO neurons with a GluR2-pHluorin^29,30^ (Fig. 7c, d). An increase in GluR2 expression at the plasma membrane has been shown to alter dendritic spine morphology, resulting in a large fraction of spines with an immature non-mushroom, filopodial appearance^31^. Such spines were abundant in the KO (Supplementary Fig. 8A-B). These data demonstrate an upregulation of surface GluR2 in syt-17 KO synapses which results in a dramatic increase in synaptic strength.

A pathological accumulation of glutamate receptors on the cell surface could be due to an upregulation in the exocytosis of these receptors, or a downregulation of receptor internalization. Given that syt-17 localizes to Rab5-positive early endosomes in neurites (Fig. 1e, Fig. 7a), and that these endosomes mediate internalization of AMPA-type glutamate receptors^32,33^, a defect in endosomal recycling seemed more likely. We observed fewer endosomes per unit dendrite (Fig. 7e, f) and a small, but significant, defect in constitutive endocytosis (Supplementary Fig. 8C) in KO neurons. Further, the expression of key endocytic proteins—including clathrin light chain, dynamin, and sorting Nexin 6—were reduced in syt-17 KO hippocampi in vivo (Supplementary Data 1).

Rab5-mediated internalization of postsynaptic AMPARs is a major mechanism underlying long-term depression (LTD) of synaptic responses in hippocampal circuits^33^. If syt-17 in fact controls synaptic strength by regulating endosomal recycling of AMPARs, then LTD, but not LTP, should be disrupted in KO animals. We measured LTD along the Shaffer collateral pathway in acute hippocampal slices prepared from WT or syt-17 KO mice, and found that this form of synaptic plasticity was abolished in slices prepared from syt-17 KO mice (Fig. 7g, h), confirming the defect in AMPAR internalization. In addition, we observed an increase in basal synaptic responses (Supplementary Fig. 8D) without an alteration in paired-pulse facilitation (Supplementary Fig. 8E), validating in slices our observation that syt-17 KOs exhibit an augmentation of synaptic strength via a postsynaptic mechanism. This alteration in synaptic plasticity is specific to LTD, as LTP was unaffected using multiple induction paradigms (Supplementary Fig. 8F-G), consistent with a role for syt-17 in the endocytosis, but not exocytosis, of AMPARs. These data collectively document a defect in endosomal recycling of AMPARs in syt-17 KO animals, leading to abnormally strong synapses, and an inability to downscale said synapses in response to circuit activity.

Syt-17 thus plays two distinct roles in neurons. One pool of syt-17 is localized to the Golgi, where it coordinates import of vesicles from the ER to the Golgi, supporting neurite outgrowth. Another pool of syt-17 is targeted to neurites, where it mediates normal endocytic recycling of AMPARs to regulate synaptic strength and plasticity.

## Discussion

While a subset of synaptotagmin isoforms have been well-characterized, most have no known function in neurons; syt-17 in particular has received scant attention. In the current study, we found that the C2 domains of syt-17 do not bind Ca^2+^ or anionic phospholipids and are unable to facilitate membrane fusion catalyzed by synaptic SNARES in vitro. We localized syt-17 in hippocampal neurons to the Golgi complex in the soma and to early endosomes in neurites. Loss of syt-17 results in impaired ER-to-Golgi trafficking, accounting for the defects in axonal and dendritic outgrowth we observed in syt-17 KO neurons. Further, a yeast two-hybrid screen revealed that separate domains of syt-17 physically interact with two resident Golgi proteins, each of which is putatively involved in tethering and import of vesicles into the Golgi from the ER^25,27^. Interaction with these two proteins is necessary for syt-17 to drive axonal outgrowth. Syt-17 thus appears to be a member of a cargo import complex on the surface of the neuronal Golgi.

Syt-17 is not ubiquitously expressed, but rather is selectively expressed in specific brain regions, so it is clearly not an essential component of the Golgi complex in most cells. Further, the molecular diversity of golgins in mammalian cells^34^ suggests a diversity of potential routes for secretory trafficking. Neurons contain multiple distinct secretory pathways^35^, including a molecularly-distinct protein translation/processing machinery that is specialized for local synthesis in dendrites^36^, and there is evidence for another specialized secretory pathway in axons^37^. We anecdotally observed that tagged syt-17 was occasionally (<1% of syt-17 puncta) colocalized with remote mannosidase-II puncta at apparent Golgi outposts in neuronal dendrites^36^, intermingled with more mobile endosomal puncta. Whether syt-17 exerts any similar modulatory effect on the trafficking of locally synthesized protein in neurites, and to what extent different secretory trafficking routes segregate distinct cargoes, are areas ripe for future study.

Mature neurons exhibit limited capacity for axonal growth, and so, when severed, the axons of mature neurons generally fail to regenerate^18^, potentially resulting in paralysis in the case of spinal cord injury. We found that overexpression of syt-17 facilitated axonal outgrowth beyond that observed in WT neurons, and reasoned that overexpression may allow mature cells to more readily regenerate following injury. Indeed, we observed that neurons overexpressing syt-17 exhibit accelerated axonal regrowth, following axotomy, in vitro. Further, this effect was specific to axons, as overexpression produced no alteration in dendritic structure. These results indicate that upregulation of syt-17 expression may have potential clinical value for spinal regeneration, a topic we will explore in a subsequent work.

In addition to facilitating ER-Golgi membrane traffic, we discovered a second function of syt-17: it regulates the strength of glutamatergic neurotransmission. Namely, syt-17 KO neurons exhibited a surprising and marked increase in synaptic transmission—contrary to the reduction in synaptic strength that may be expected from a defect in secretory trafficking^22^. This increase was also detectible in hippocampal slices. After ruling out several possible mechanisms, we found that this phenotype has a postsynaptic locus, and is attributable to accumulation of excess AMPA-type glutamate receptors on the dendritic surface. This accumulation appears to alter dendritic spine morphology, resulting in more filopodia and few spines^31^. However, we cannot formally rule-out the possibility that these changes in spine shape or other synaptic phenotypes are somehow secondary to dysfunction in the secretory pathway. Novel tools to selectively delete proteins from specific intracellular compartments are needed to fully disentangle these two possibilities and confirm that the role of syt-17 in the phenotypes reported here is direct. We further observed that syt-17 KO neurons exhibit a deficiency in endosomal recycling. We reiterate that the pool of syt-17 that is expressed at or near synapses localizes to early endosomes, and endocytosis of surface AMPA-type receptors is mediated by these early endosomes^33^. We therefore proposed that the accumulation of excess glutamate receptors in the plasma membrane of dendrites in syt-17 KO neurons is due to a deficiency in Rab5-dependent early endosomal trafficking. Further, dysregulation of this process in syt-17 KO animals is associated with a specific deficit in LTD of synaptic responses, potentially providing an explanation for the observed hippocampal-dependent memory deficits in these animals.

In summary, syt-17 coordinates efficient Golgi import in the early secretory pathway to control neurite outgrowth, and it regulates the trafficking of early endosomes in dendrites to control the surface density of AMPA receptors. Syt-17 appears to be one shared element of neuron-specific adaptations in the secretory and endosomal systems, and dysregulation of either of these processes has profound effects on neuronal development and physiology. Syt-17 is therefore unusual among the syt family of proteins, as it executes two distinct, Ca^2+^-independent, functions in intracellular membrane trafficking that have no precedent in this protein family. A relatively small number of synaptotagmin isoforms (chiefly syt-1 and syt-7) have been the focus of intense study, and that body of research has overwhelmingly focused on explicating their role in Ca^2+^-triggered exocytosis. The present work suggests an untapped diversity of roles for more exotic synaptotagmins in neuronal and synaptic biology.

## Methods

### Ethics statement

All animal experiments were conducted at the University of Wisconsin-Madison, and protocols were reviewed, and received approval, by the university’s Animal Care and Use Committee (assurance # A3368-01). All relevant ethics regulations for animal testing and research were followed, and the guidelines set by the NIH *Guide for the Care and Use of Laboratory Animals* handbook were conformed to in all cases.

### Recombinant proteins and protein purification

Constructs encoding syt-1 C2AB (a.a. 96–421) and syt-17 C2AB (a.a. 152–474) were expressed as GST fusion proteins (pGEX-4T vector, GE) in *E. coli*, purified via glutathione-Sepharose affinity chromatography, and cleaved with thrombin in 100 mM KCl, 25 mM HEPES pH 7.4, 5% glycerol. HaloTag constructs were assembled by overlap extension PCR and subcloned into pTrcHis A vector (ThermoFisher) to yield N-terminal His_6_-HaloTag-syt constructs. These constructs were expressed in *E. coli*, purified via nickel-NTA chromatography, and eluted in His_g_-tag elution buffer (500 mM imidazole, 400 mM KCl, 25 mM HEPES pH 7.4, and 10% glycerol). Full-length syt-17 was purified by affinity chromatography of *E. coli* lysates using HaloLink resin (Promega) and eluted by cleavage with TEV protease in 100 mM KCl, 25 mM HEPES pH 7.4, 5% glycerol, 1% Triton X-100 with 2 mM DTT.

### Liposome preparation (fusion assays)

The following lipids were purchased from Avanti Polar Lipids: 1-palmitoyl-2-oleoyl-*sn*-glycero-3-phosphoethanolamine [phosphatidylethanolamine (PE)]; 1,2-dioleoyl-*sn*-glycero-3-phospho-l-serine [phosphatidylserine (PS)]; 1-palmitoyl-2-oleoyl-*sn*-glycero-3-phosphocholine [phosphatidylcholine (PC)]; 1,2-dipalmitoyl-*sn*-glycero-3-phospho-ethanolamine-*N*-(7-nitro-2–1,3-benzoxadiazol-4-yl) (NBD-PE); *N*-(lissamine rhodamine B sulfonyl)-1,2-dipalmitoyl-*sn*-glycero-3-phosphoethanolamine (rhodamine-PE); 1,2-dioleoyl-sn-glycero-3-phosphoethanolamine-*N*-(5-dimethylamino-1-naphthalenesulfonyl) (dansyl-PE). *N*,*N*′-dimethyl-*N*-(iodoacetyl)-*N*′-(7-nitrobenz-2-oxa-1,3-diazol-4-yl)ethylenediamine (IANBD-amide) was purchased from Invitrogen. v-SNAREs were reconstituted into vesicles containing membrane-bound FRET (fluorescence resonance energy transfer) donor-acceptor pairs, and t-SNARES were reconstituted into unlabeled vesicles^38^.

### In vitro fusion assays

Lipid compositions for the vesicles used in the in vitro fusion assays: 15% PS, 27% PE, 55% PC, 1.5% NBD-PE, and 1.5% Rhodamine-PE for synaptobrevin 2 (syb2) vesicles; 15% PS, 30% PE, and 55% PC for heterodimer syntaxin1a/SNAP-25B vesicles.

Fusion between syb2 vesicles and heterodimer vesicles was monitored using a Synergy HT multidetection microplate reader (Bio-Tek)^38,39^. For the protein titration 0–10 μm of syt-1 C2AB or syt-17 C2AB was used. During each run, 1 mM final free Ca^2+^ was added at 20 min, and the reaction was monitored for an additional 120 min. Traces were normalized to the first timepoint and the maximum fluorescence signal, determined from the addition of *n*-dodecyl-β-d-maltoside, to determine the %*F*_max_.

### Isothermal titration calorimetry (ITC)

Syt-1 C2AB and syt-17 C2AB were dialyzed overnight against 50 mM HEPES-NaOH (pH 7.4), 200 mM NaCl, and 10% glycerol; in order to remove divalent cations, the buffer was pre-treated with Chelex-100 resin (Bio-Rad). Filtered dialysis buffer was used to make all protein and Ca^2+^ dilutions. Samples were degassed before each experiment. Heat of binding was measured by 20 consecutive injections of Ca^2+^ into a sample cell containing the protein of interest. Corrections for heat of dilution were done by subtracting the signal of Ca^2+^ into buffer. Experiments were performed using a MicroCal iTC_200_ (Malvern Instruments).

### Liposome preparation (cosedimentation assays)

Liposomes were prepared from POPC, DOPS, POPE, 18:1 ceramide, and brain PIP_2_ (all from Avanti Polar Lipids) stored individually as chloroform stocks, except for brain PIP_2_ (stored in 20:9:1 CHCl_3_:MeOH:H_2_O). Liposome compositions for cosedimentations were as follows: PC:PE: 70% PC, 30% PE; + PS: 45% PC, 30% PE, 25% PS; + PS + PIP_2_: 44% PC, 30% PE, 25% PS, 1% PIP_2_; + cer: 55% PC, 25% PE, 5% PS, 15% ceramide. The lipids were combined, the solvent was evaporated under a stream of nitrogen, and the films were dried under vacuum for at least 2 h. Films were rehydrated in reconstitution buffer (100 mM KCl, 25 mM HEPES pH 7.4) at a final concentration of 10 mM [lipid] and extruded at least 29 times through a single 100-nm polycarbonate filter (Whatman).

### Equilibrium cosedimentation assays

Liposomes (2 mM lipid), C2AB (4 μΜ), and EGTA (0.5 mM) were combined and brought up to 100 µl in reconstitution buffer with or without 1.5 mM CaCl_2_ added. The mixture was incubated for 15 min at room temperature with shaking, loaded into a polycarbonate centrifuge tube, and centrifuged at 160,000×*g* for 30 min in a TLA-100 rotor (Beckman). An aliquot of the supernatant was combined 1:1 with 2× SDS sample buffer and subjected to SDS-PAGE. Gels were stained with Coomassie blue and the bands quantified by densitometry.

### Cell culture and transfection

The syt-17 KO mouse employed in this study was generated from ES cell clone EPD0659_3_A09, acquired from the KOMP repository (www.komp.org) by the Wellcome Trust Sanger Institute (WTSI). WTSI and the Children’s Hospital Oakland Research Institute generated the targeting vectors as part of the KO Mouse Project (3U01HG004080; methods described previously^40^). Animals were group-housed in the Wisconsin Institute for Medical Research vivarium at UW-Madison.

Primary hippocampal neurons (CA subfields) were harvested from newborn (P0) mice of both sexes and cultured^41^. Hippocampi (CA subfields) from each pup in a litter were dissected and incubated in 0.25% trypsin-EDTA (Corning), 20 mM D-Glc, and 25 U/ml DNase for 22 min. Tissue was washed twice, mechanically dissociated, and plated on poly-D-lysine (Life Technologies) coated glass coverslips in a solution of Dulbecco’s Modified Eagle Medium (Gibco) with 10% fetal bovine serum. Once cells attached (<1 h), media was changed to a growth media consisting of Neurobasal-A (GIBCO) supplemented with 2% B27 (GIBCO) and 2 mM Glutamax (Invitrogen). Cultures were maintained in a 5% CO_2_-humidified incubator at 37 °C; 1/3 of the media volume was replaced every three days, as we have found this feeding scheme optimal for neuronal health. All other reagents were purchased from Sigma except as indicated below.

Most transfections were performed using the Ca^2+^-phosphate method^42^ at 3 DIV. For axonal regrowth experiments, postnatal mouse or E18 rat neurons were seeded in microfluidic devices (SND450, Xona Microfluidics) at a density of 55-65k cells per chamber. For experiments imaging fluorescent proteins in young (2–4 DIV) neurons, transfection was performed with a Neon (Invitrogen) electroporation system according to the manufacturer’s instructions. For axon regeneration experiments, cells were transduced at 9 DIV with HaloTag-Syt-17_eGFP lentivirus, or a control lentivirus containing HaloTag-eGFP. For experiments using VSVG-YFP-2xUVR8, transfections were performed using Lipofectamine LTX (ThermoFisher) at 6DIV (24–36 h before the experiment) according to the manufacturer’s instructions, as prolonged expression of this probe results in leak to the plasma membrane.

### RT-PCR

Successful knockout was verified with RT-PCR from brain lysates of six-week-old animals. Three separate sets of probes against syt-17 were used. 1F: AATCCAGCTGGTACACGGACTCAA, 1R: ACACTGTGAATACTAGGCTGGCGT, 2F: GCCAGTCCAGTGAAGATGAA, 2R: GATTGGAGTCGAGGGAGTAAAG, 3F: GAACGAGGGCTTGCTTTCTA, 3R: GCCAGCACTTGGGAGATAAT. Probes directed against β-actin were used as controls, and values were converted to a ratio of KO expression as a percentage of WT.

### Mouse behavior

Six-to-eight-week-old male and female mice were used for behavioral characterization. Tests were conducted in the Waisman Rodent Behavior core or the Wisconsin Institute for Medical Research vivarium at the University of Wisconsin-Madison. The experimenter was blind to genotype during testing and all photographic/video analysis. Animals were given a minimum of 24 h between tests, and were moved to the testing rooms at least 30 min prior to the assay for acclimation. First, each mouse received a single 30 min open field exploration session. Each mouse was removed from its home cage and placed in the center of the arena, where the Omnitech Fusion system used photobeams to continuously monitor and record the animal’s placement during the assay. Data was recorded using the Fusion system with a center ratio zone map.

For the marble burying test, each mouse was tested in a clean home cage that had been filled to a depth of 4 cm of fresh bedding. Twenty marbles were placed on top of the bedding in a grid arrangement (see below). Mice were placed into the test cages and allowed to explore for 30 min. Following the completion of the test, mice were returned to their home cages. The number of marbles that were at least 50% buried was recorded. Photographs were taken, from a vantage point directly above each cage, to show position and arrangement of the marbles.

For the novel object recognition task, animals were placed in a chamber with bedding, containing two identical unfamiliar objects spaced approximately six inches apart, and were permitted to explore for 10 min. Following a twenty-four-hour interval, animals were returned to the same chamber, but one of the familiar objects had been replaced with a novel object. Animals were again permitted to explore the chamber and objects and ten minutes. Behavior during familiarization and testing phases were videotaped for offline analysis. The objects were Fisher-Price Little People Batman and Superman figures, or wooden toy police cars (which was treated as novel and which as familiar was counterbalanced across animals). The chamber and object were thoroughly cleaned between animals/trials to remove olfactory cues. Behavior during familiarization and testing phases were videotaped for offline analysis, and a Discrimination Index^43^ was computed.

All mice were tested in the social interaction paradigm, a 3-trial assay with each trial lasting 10 min. During the initial acclimation trial, the mouse was placed inside of the three-chambered box and allowed to explore freely with no objects presented. The mouse was removed, the arena was cleaned and prepared, and the mouse was returned to the arena for the sociability test. During the sociability test, a male juvenile mouse was placed in a mesh cup in one chamber of the box and an identical empty mesh cup was placed in the opposite chamber of the box to provide a neutral object control. The cups were weighted to prevent mice from moving them inside the arena. At the conclusion of the sociability test, the mouse was removed, the arena cleaned and prepared, and the mouse returned to the arena for the social recognition test. During the recognition trial, the juvenile stimulus mouse from the sociability test was kept in the test arena, and a novel male juvenile mouse was placed in a cup in the opposite chamber. The sociability and recognition trials were videotaped for subsequent analysis. Locations of the stimuli mice were counterbalanced. Videotapes were analyzed for interaction behavior. Interaction behavior included sniffing, biting at the cup, pawing, or other object-directed behavior. Climbing on the cup that did not involve sniffing the stimulus mouse inside the cup was not considered interaction behavior. Time spent engaged in investigative behavior for each cup was measured (in seconds), total investigative time calculated, and percent preference scores obtained.

Finally, animals were subject to tone/shock fear conditioning. On day 1, animals were subject to a six-minute training phase, which consisted of 2 pairings of 30 s of 87 dB white noise and a 1.5-s 0.7 mA shock, with an intertrial interval of 2 min. Twenty-four hours later, mice were tested for conditioning to the tone in a novel chamber, by altering the chamber with plexiglass inserts and vanilla extract. These changes provide a novel environment so that response to the cue was minimally affected by any conditioning to context. Mice were placed back in the chambers for a 6 min test and the noise was played for the final 3 min of the test. Percent freezing during each portion of the assay was calculated by the FreezeFrame 3 software using video feed and a motion index.

### Live-cell imaging and analysis

Measurements of axonal outgrowth were performed at 2–4 DIV on an Olympus CellTIRF with DIC optics using a ×60 Apo N objective and Hamamatsu Orca-FLASH 4.0 camera. Only cells that were identifiably stage 3 (extending an axon at least 2–3× longer than other neurites), with visible growth cones, whose axons were not growing along processes of other cells, were selected for imaging. Neurons were maintained in their native growth media in an environmentally controlled chamber with 5% CO_2_ at 37 °C; typical imaging duration was 8–12 h (1 min capture interval). Outgrowth rate, growth cone area, and spontaneous growth cone collapse were measured offline in MetaMorph software (Molecular Devices). Live-cell imaging of overexpressed fusion proteins was conducted similarly, at ages indicated in the text. Localization of pHluorin-tagged syt-17 was performed, in parallel, with constructs tagged at both the N-termini and C-termini, to increase confidence that the protein was not mis-sorted and that both ends of the protein are located in the cytosol.

To measure retrograde actin flow, we incubated stage 3 cultured hippocampal neurons prior to imaging for 3 min in 100 pM of SiTMR-KabC, a fluorescently labeled kabiramide^44^ kindly gifted by Gerard Marriott (University of California-Berkeley), which at low concentrations selectively labels the barbed ends of actin filaments. Retrograde flow rates were quantified by computing a kymograph in ImageJ from 2–3 filopodia per growth cone, fitting three lines per kymograph, and averaging the resultant values within a growth cone^45^.

For measurements of axonal regeneration, axotomy was performed at DIV 14–15 by rapid aspiration and reperfusion of media from the axon channel. Conditioned media was removed from the axon side of the microfluidic prior to axotomy and replaced post-axotomy. Axons were labeled with Vybrant DiI Cell-Labeling solution (5 nM) on the axon side for two hours and rinsed with media prior to imaging. Images were taken prior to axotomy and immediately after to establish a baseline, and again both 24 and 48 h after axotomy to measure regrowth.

Internalization of fluorescently labeled transferrin-546 (25 µg/mL, ThermoFisher) was monitored on an upright Olympus FV1000 confocal laser-scanning confocal microscope with a ×60 LUMFL water immersion objective in an environmentally-controlled chamber.

Measurements of ER-to-Golgi trafficking using VSVG-YFP-2xUVR8 were performed on an Olympus IX81 inverted microscope with a Lambda DG-4 light source, Olympus ×60 Plan Apo N objective, and Hamamatsu Orca-FLASH 4.0 camera. Z-series were collected every 30 s for 40 min at 7–8 DIV (pilot experiments showed this to be the optimal window for expression). For uncaging, a 300 nm fiber-coupled LED (ThorLabs M300F2) was positioned ~0.5 cm above the cell media, and the sample was illuminated for 10 s. Offline, a small ROI was drawn in the Golgi and the timecourse of cargo accumulation was quantified for each cell.

For Ca^2+^ imaging, 13–15 DIV neurons were depolarized with 40 mM KCl and loaded with 14.8 µM FM-464 (Thermo Scientific) for 10 min to label synaptic boutons. Cells were washed with artificial cerebrospinal fluid (ACSF) containing 128 mM NaCl, 5 mM KCl, 2 mM CaCl_2_, 1 mM MgCl_2_, 30 mM Glc, and 25 mM HEPES (pH 7.4, mOsm 310). For this wash step, ACSF was supplemented with 1 mM ADVASEP-7 (Sigma). Cells were then loaded with 13.6 µM Fluo-5F AM (with 1% Pluronic F-127, Thermo Scientific) for a further 10 min, washed, and transferred to a field stimulation chamber. Imaging was performed on an Olympus CellTIRF with a ×60 Apo N objective. Imaging fields of view were selected to maximize the number of boutons (visualized by FM4-64) on isolated processes, taking care to avoid glia (identifiable by morphology, kinetics of evoked Ca^2+^ responses, and/or high resting Ca^2+^ signal). Images were acquired 100 Hz with 2 × 2 pixel binning (482 nm excitation). During imaging, 50 µM D-APV (Abcam), 100 µm picrotoxin (Tocris), and 10 µm CNQX were included in the ACSF. Atypically-large FM-464 puncta (likely representing either endosomes or closely adjacent boutons) were excluded from analysis. Ca^2+^ responses were quantified from individual boutons following a single action potential, converted to ΔF/F_0_ (change in fluorescence divided by baseline fluorescence), and the peak of each response was extracted. For every imaging field of view, the baseline fluorescence of our synaptic ROIs was significantly greater than background (i.e., areas of the coverslip without cells) fluorescence, and this baseline fluorescence did not differ between genotypes (*p* = 0.9), justifying the Δ*F*/*F*_0_ normalization.

Imaging of GluR2-pHluorin (Addgene plasmid #24001) was performed on the same CellTIRF setup as described above, substituting 0.5 μM tetrodotoxin (TTX, Abcam) for APV/CNQX/picrotoxin in the ACSF. Soluble mRuby2 was cotransfected with the pHluorin constructs to visualize dendritic morphology. To determine the surface expression level, cells were alternatively perfused with acidic ACSF (25 mM N-morpholino] ethane sulfonic acid substituted for HEPES, pH 5.5) or ACSF with ammonium chloride (50 mM NH_4_Cl and 38 mM NaCl). ROIs were selected on dendrites and spines (visible as puncta in during NH_4_Cl perfusion), and surface expression was calculated as ((pH 7.4 fluorescence–pH 5.5 fluorescence)/(NH_4_Cl fluorescence–pH 5.5 fluorescence)) × 100^46^. Greater > 10 synapses across multiple dendrites were measured for each cell.

### Fixed-cell imaging and analysis

Morphological analysis of axon lengths, dendritic arbors, and spine morphology were performed by sparsely-transfecting cells with GFP, fixing the cells in 4% paraformaldehyde at the appropriate DIV (7 DIV for axons, 14–15 DIV for dendrites and spines), and immunostaining for the GFP (Abcam). This allowed full reconstruction of transfected cells. For most measurements, immunostaining against MAP2 was also performed to ensure accurate identification of axons and dendrites. Coverslips were imaged on an Olympus FV1000 confocal microscope with a ×20 XLUMPlanFL N, ×60 PlanApo N, or ×100 UApo N objective. Resultant neurite arbors were reconstructed in ImageJ and Sholl analysis^47^ was performed.

### Electron microscopy

Sapphire discs (3 mm) were washed in acetone and subsequently in 95% ethanol. Discs were coated first with carbon followed by gold and baked overnight at 160 °C. Discs were then plasma glow discharged, poly-L-lysine coated overnight at 37 °C, and then coated with laminin for 2–3 h. Discs were UV sterilized, and hippocampal neurons were plated at a density of 50–100,000 cells/cm^2^. Neurons at 3 and 15 DIV were rapidly frozen under high pressure in a Wohlwend Compact 02 High-Pressure Freezer (Engineering Office M. Wohlwend GmbH CH-9466 Sennwald/Switzerland) and freeze substituted into acetone containing 2% osmium tetraoxide and 0.1% uranyl acetate at −80 °C, then slowly warmed to room temperature and embedded in EPON-Araldite. Three hundred nm thick sections of embedded neurons were cut and stained using 2% uranyl acetate and lead citrate. Additional details on sample preparation have been described elsewhere^48^. Imaging was performed using a Tecnai F30 (FEI, Eindhoven, The Netherlands) operated at 300 kV and nominal magnification of ×20,000, and samples were tilted from 60° to −60° at 1.5° increments along a dual axis. A total of 80 images with a pixel size of 1.0194 nm were collected with CCD camera (Gatan, One View 4 K × 4 K pixels). Images were processed using 3dmod, version 4.9.0 software package^49,50^.

### Yeast two-hybrid

DEEPN (Dynamic Enrichment for Evaluation of Protein Networks), a method for performing comparative Yeast two-hybrid assays in batch^51–53^, was conducted. An initial screen of syt-17 mutants found that a construct lacking the C2A domain expressed at high levels, and was therefore used for initial testing. Briefly, syt-17 ΔC2A was cloned in frame to the Gal4-DNA-binding domain housed in the TRP1-containing plasmid pGBKT7, which was subsequently transformed into PJ69-4A MATA cells^54^ and mated with PLY5725 MATalpha cells carrying a library of fragments derived from the human ORFeome^55^ housed in the LEU2-containing plasmid pGAL4-AD. Two additional library containing populations were made using empty pGBKT7. Yeast populations were divided and grown in the presence and absence of histidine to produce subpopulations that had not or had been selected based on their ability to produce a positive yeast two-hybrid interaction, respectively. After growth, library fragments were amplified by PCR and 1–2 × 10^7^, 2 × 150 PE reads were obtained per sample on an Illumina HiSeq 4000. Reads were mapped to the hg38 genome using HiSTAT2 with the Mapster interface, and further analyzed using the DEEPN and statistics software^53^.

For binary two-hybrid assays, yeast lysates from cells transformed with pGal4-AD plasmids containing GOLGA6A and ICA1 fragments corresponding to the regions delineated from DEEPN sequence data, were immunoblotted (anti-HA) for expression of the HA-tagged AD-fusion proteins. The pGal4-AD plasmids containing GOLGA6A or ICA1 were transformed into PLY5725, mated to PJ69-4A cells carrying the pGBKT7 vector alone or syt-17 plasmids, and plated onto minimal media with and without Histidine to measure binary interactions. In certain incubations (indicated in figure legends), 1 mM or 10 mM of 3-aminitriazole was included to increase the stringency of selection.

### HaloTag SNARE binding assays

Purified Halo-tagged constructs (100 µg) were combined with HaloLink resin (100 µl bed volume), and the mixture was brought up to 500 µl with binding buffer (150 mM KCl, 25 mM HEPES pH 7.4, 1% Triton X-100, 1 mM DTT) and incubated 30 min at room temperature with rotation. Complete depletion of Halo-C2AB from the supernatant under these conditions was verified by SDS-PAGE. Beads were washed 3× in binding buffer and stored for no longer than 4 days at 4 °C.

For pulldowns from brain lysates, a single 3–4 week old C57/BL6 mouse was euthanized with CO2, decapitated, and its whole brain removed. The brain was transferred into 5 ml ice-cold lysis buffer (150 mM NaCl, 50 mM HEPES-NaOH pH 7.4) with protease inhibitors added (cOmplete mini, Sigma) and subjected to 12 strokes in a Teflon-glass Dounce homogenizer rotating at 900 RPM. The crude homogenate was assayed for protein content, diluted to 1 mg/ml protein, and combined with Triton X-100 (2% final concentration; 2:1 detergent:protein ratio). This mixture was incubated for 30 min with rotation at 4 °C and clarified by centrifugation (3500×*g*, 10 min). A 50% slurry of protein-bound HaloLink resin (90 μl, 45 μg bait protein) was added to 6 ml of clarified lysate, and the mixture incubated for 3 h at 4 °C with rotation. The beads were then collected, washed 3× with 1 ml lysis buffer containing 1% Triton X-100, and eluted in 60 µl 4× SDS sample buffer containing 1% Triton X-100. The input lysate and eluates were assayed by Western blot using primary antibodies to syntaxin-5 or syntaxin-13 (both from Synaptic Systems) with incubation overnight at 4 °C followed by detection with goat anti-rabbit IgG-HRP secondary antibody (Abcam).

### Mass spectrometry

Hippocampi from six-week old syt-17 WT (four animals) and KO (four animals) from separate breeding pairs were harvested and homogenized in 6 M Guanidine, 50 mM HEPES (pH 8.5) in Precellys 24 (Program #2). Cysteines were alkylated with iodoacetamide (IAA, to a final concentration of 15 mM) and incubated 20 min at room temperature in the dark. Excess IAA was quenched with Dithiothreitol (DTT) for 15 min. Samples were diluted with 200 mM HEPES pH 8.5 to 1.5 M Guanidine, followed by digestion at room temperature for 3 h with LysC protease at a 1:100 protease-to-protein ratio. Following LysC digestion, trypsin was added at a 1:100 protease-to-protein ratio followed by overnight incubation at 37 °C with shaking. The reaction was quenched with 2% formic acid, desalted using C18 solid-phase extraction (HyperSep, Thermo Scientific), and vacuum centrifuged to dry. For TMT labeling, desalted peptides were dissolved in Triethylamonium bicarbonate (TAEB) solution. Peptide concentration was measured by μBCA (Pierce), and 100 μg of peptide per sample were labeled with TMT reagents (final anhydrous acetonitrile concentration of 30% (v/v)). Samples were labeled as follows: WT_1 (TMT 127 N); WT_2 (TMT 127 C); WT_3 (TMT 128 N); WT_4 (TMT 128 C); KO_1 (TMT 129 N); KO_2 (TMT 129 C); KO_3 (TMT 130 N); KO_4 (TMT 130 C). Following incubation at room temperature for 75 min, the reaction was quenched with hydroxylamine to a final concentration of 0.5% (v/v). TMT-labeled samples were combined at a 1:1:1:1:1:1:1:1 ratio, vacuum-centrifuged to near dryness, subjected to High pH Reversed-Phase Peptide Fractionation (Pierce), followed by C18 extraction (Pierce), and vacuum centrifugation to dryness.

Three micrograms of each sample was auto-sampler loaded with a Thermo RSLC UPLC pump onto a vented Acclaim Pepmap 100, 75 µm × 2 cm, nanoViper trap column coupled to a nanoViper analytical column (cat. #: 164570, Thermo, 3 µm, 100 Å, C18, 0.075 mm, 500 mm) with stainless steel emitter tip assembled on the Nanospray Flex Ion Source with a spray voltage of 2000 V. A coupled Orbitrap Fusion (Thermo Fisher Scientific) was used to generate MS data. Buffer A contained 94.785% H_2_O with 5% acetonitrile and 0.125% formic acid, and buffer B contained 99.875% acetonitrile with 0.125% formic acid. MultiNotchMS3-based TMT method was used for TMT samples1–3. The scan sequence began with an MS1 spectrum (Orbitrap analysis, resolution 120,000, 400–1400 Th, AGC target ^2 × 105^, maximum injection time 200 ms). MS2 analysis, ‘Top speed’ (2 s), Collision-induced dissociation (quadrupole ion trap analysis, AGC ^4 × 103^, NCE 35, maximum injection time 150 ms). MS3 analysis, top ten precursors, fragmented by HCD prior to Orbitrap analysis (NCE 55, max AGC ^5 × 104^, maximum injection time 250 ms, isolation specificity 0.5 Th, resolution 60,000).

Protein identification and quantification analysis were done with Integrated Proteomics Pipeline (Integrated Proteomics Applications, Inc. San Diego, CA) using ProLuCID, DTASelect2 and Census. Tandem mass spectra were extracted into ms1, ms2 files, and ms3 from raw files using RawConverter (http://fields.scripps.edu/downloads.php) and were searched against UniProt mouse protein database (released on 03-25-2014), matched to sequences using the ProLuCID/SEQUEST algorithm, and filtered with DTASelect2. Searches were performed using a 50 ppm precursor ion tolerance, 600 ppm fragment ions, and included all fully-tryptic and half-tryptic peptide candidates with no missed cleavages restriction. Protein false-discovery rate (FDR) was set to 0.01. Carbamidomethylation (+57.02146) of cysteine and N-termini lysine (+229.1629) were considered as static modifications. Resulting data was quantitated using software Census with batch-specific correction factors (TMT 10-plex lot no. RC231246), with intensity threshold set to 10,000. Statistical overrepresentation tests of gene ontology (GO) terms were performed with PANTHER gene analysis tools^56^ using UniProt accession numbers of canonical isoforms as inputs and Bonferroni correction for multiple testing.

### Electrophysiology

Whole-cell patch-clamp recordings were acquired from 13–16 DIV neurons using a MultiClamp 700b amplifier and pClamp software (Molecular Devices). Patch pipettes (3–5 MΩ resistance) were filled with an intracellular solution containing 135 mM cesium-methylsulfate, 5 mM KCl, 2 mM NaCl, 0.2 mM EGTA, 10 mM HEPES, 10 mM Na_2_-phosphocreatine, 5 mM MgATP, 0.3 mM Na_2_-GTP. Five mM QX-314 was included in the patch pipette for evoked EPSC recordings to prevent depolarization of the postsynaptic neuron. The recording chamber was continually perfused with an ACSF containing 128 mM NaCl, 5 mM KCl, 2 mM CaCl_2_, 1 mM MgCl_2_, 30 mM D-Glc, 25 mM HEPES, 50 µm APV, and 100 µm picrotoxin (pH 7.4, mOsm 310). Postsynaptic (putative pyramidal) neurons were clamped at −70 mV. Measurements were aborted if a series resistance >15 MΩ was observed, or if the series resistance changed >10% during recording.

For evoked EPSC recordings, a bipolar electrode in theta glass was positioned at the soma of a neuron adjacent to the patched cell and a single biphasic pulse was applied. The stimulation voltage (using a Warner A350 stimulus isolator) was set at 5 V and gradually increased/decreased to ensure the resulting response was unitary. EPSCs that lacked a smooth rising phase (potentially suggesting disynaptic excitation) were excluded from analysis. For measurement of mEPSCs, 0.5 µm tetrodotoxin was added to the bath and spontaneous quantal currents were recorded for >3 min. Miniature events were identified using a template-matching algorithm in Clampfit.

For glutamate puffing experiments, neurons were first filled with 0.2% of Alexa 488 Biocytin (ThermoFisher) in intracellular solution through the patch pipette to visualize morphology. Only neurons with an obvious pyramidal-like morphology (possessing an elliptical soma, visible dendritic spines, a clear main/apical dendrite, and some number of small basal dendrites) were stimulated. Subsequently, a second patch pipette containing ACSF with 200 µm L-glutamate and 200 µm Alexa 488 Hydrazide (ThermoFisher) was positioned along the main (apical) dendrite ~60 µm away from the soma. Fluorescence from the stimulation pipette and membrane current in patch cell were monitored during approach to ensure minimal leak. Pulses of glutamate were then applied for 5 ms with a Picospritzer III (Parker) as the cell holding voltage was varied from −70 to +40 mV in 22 mV increments. The ejection of the glutamate/dye was visually monitored during the experiment. The stimulation pipette was moved closer to the dendrite each trial. When the pipette was moved close enough to rupture the cell (and the patch was lost), the previous trial was selected for analysis. The peak current amplitude was quantified for analysis. Note that in this experimental configuration, the decay from peak of a given response can be affected by the vagaries of dendritic geometry and ACSF flow; as such, decay kinetics of these responses are not interpretable, and were not analyzed. Measurements for all experiments were made from ≥3 independent preparations.

### Slice recordings: chemical LTD

Measurements of long-term depression (LTD) were performed on acute hippocampal slices prepared from adult littermate male mice (6 WT, 8 KO, 6–7 weeks old). Hippocampal extractions were performed at the same time of day for every experiment. After decapitation, the brain was rapidly extracted and placed in a frozen slurry of cutting solution (CS; 0.6 mM sodium ascorbate, 3 mM KCl, 1.25 mM NaH_2_PO_4_, 60 mM NaCl, 28 mM NaHCO_3_, 7 mM MgCl_2_, 0.5 mM CaCl_2_, 5 mM D-glucose, and 110 mM sucrose). Horizontal sections of the hippocampus (400 µm) were isolated using a Vibratome while submerged in the CS slurry. After sectioning, slices recovered at room temperature for 45 min in a solution containing 50% CS and 50% artificial cerebrospinal fluid (ACSF; 2.5 mM KCl, 1.25 mM NaH_2_PO_4_, 125 mM NaCl, 25 mM NaHCO_3_, 1 mM MgCl_2_, 2.5 mM CaCl_2_, and 25 mM D-glucose]. Slices were then placed in a beaker containing 100% ACSF at room temperature for an additional 45 min. After recovery, slices were transferred to an interface chamber (Fine Science Tools, Foster City, CA). Hippocampal slices were equilibrated on the recording rigs for 2 h while being perfused with ACSF warmed to 32 °C (TC-324B, Warner Instrument Corporation, Hamden, CT) at a rate of 1.5 ml/min using a peristaltic pump. All solutions used in the presence of live tissue were constantly carb-oxygenated (95% O_2_/5% CO_2_).

Bipolar stimulating electrodes were made using isonel enameled platinum-tungsten wire (92:8 Pt:Y; Sigmund Cohn Corporation, Mt. Vernon, NY) and placed on the Schaeffer collateral axon bundles extending from the CA3 to CA1. Recording electrodes were made from single barrel borosilicate capillary glass pipettes with microfilaments (A-M Systems). The electrodes were filled with ACSF (4–5 MΩ) and placed on the CA1 *stratum radiatum*. Baseline synaptic transmission was evaluated by measuring input:output relationship (0.5V-20 V, 25 nA-2 µA, using A-M Systems model 2200 stimulus isolator). Subsequent stimulations for the slice were set at 50% the maximum field excitatory post synaptic potential (fEPSP) slope elicited from the input:output paradigm.

Slices were stimulated every 20 s, and the average of 2 min sweeps were used to generate a single data point. The fEPSPs were amplified (A-M systems model 1800) and digitized (100 kHz, Digidata 1322B, Molecular Devices) prior to being analyzed (pClamp, Molecular Devices). Graphical representations of the data were generated by measuring post induction fEPSP slopes and normalizing them to the average fEPSP slope at baseline (60 min prior to LTP or LTD induction). Slices with unstable baselines (>10% deviation from across the baseline) were not used in final data analysis. NMDAR-LTD was induced by perfusing 20 µM NMDA (Tocris) over hippocampal slices for 3 min^13^. Following bath application of NMDA, slices were bathed in ACSF. Time 0 for LTD was deemed at the point when the evoked fEPSP responses fell below the average baseline response by greater than 10%. NMDAR-LTD was recorded for 90 min following the initial depression of evoked fEPSP responses.

### Slice recordings: LTP

Long term potentiation (LTP) was measured in vitro from hippocampal slices of Syt-17 KO and WT mice. Adult littermate male mice (5 WT, 7 KO, 6–10 weeks old) were anesthetized with isoflurane and decapitated. The brain was rapidly removed and placed in a frozen slurry of cutting solution (124 mM NaCl, 1.25 mM NaH_2_PO_4_, 3 mM KCl, 25 mM NaHCO_3_, 10 mM glucose, 1 mM sodium ascorbate, 3 mM kynurenic acid, 3.6 mM MgSO_4_, and 0.8 mM CaCl_2_, bubbled with a carbogen mixture of 95% O_2_/5% CO_2_). Coronal hippocampal slices (400 μm) were cut using a Vibratome (Campden Instruments model 7000smz2) and allowed to recover in artificial cerebral spinal fluid (aCSF; 124 mM NaCl, 1.25 mM NaH_2_PO_4_, 3 mM KCl, 25 mM NaHCO_3_, 15 mM glucose, 0.8 mM sodium ascorbate, 1.3 mM MgSO_4_, and 2.5 mM CaCl_2_, bubbled with a mixture of 95% O_2_/5% CO_2_) at an elevated temperature (37 °C). After 30 min, slices were recovered in aCSF at room temperature for an additional 60 min. Individual slices were placed in submerged recording chambers and perfused with aCSF using a peristaltic pump (Gilson model MINIPULS3) at a rate of 3 mL/min. All recordings were taken at 30 °C. A concentric bipolar stimulating electrode (World Precision Instruments) driven by a stimulus isolator (Multi Channel Systems model STG4004) was used to stimulate the Schaffer collateral pathway every 20 s. Field excitatory post-synaptic potentials (fEPSPs) were recorded from the stratum radiatum of the CA1 hippocampal region using tungsten microelectrodes (0.1MΩ, World Precision Instruments). The stimulation intensity was set below the population spike threshold for each slice, at 50% of the maximum fEPSP slope as determined by a input:output curve performed at the beginning of each experiment. fEPSP slopes were visualized and analyzed using WinLTP synaptic electrophysiology software (WinLTP Software, Bristol, UK).

LTP was induced in stable slices (baseline deviation < 10% over 30 min) using theta-burst stimuli (TBS). In one set of experiments, the TBS paradigm consisted of one train of 10 bursts, with each burst consisting of 4 pulses at 100 Hz, delivered every 200 ms (TBSx1). In the second set of experiments, the paradigm consisted of three trains of 10 bursts, with trains delivered every 20 s (TBSx3). LTP was recorded for 60 min following TBS. For each slice, potentiation was defined as the average fEPSP slope during the last 10 min divided by the baseline fEPSP slope (average of the 10 min immediately preceding TBS).

### Statistical analysis

Image analysis was performed in ImageJ/FIJI^57^ and MATLAB (Mathworks), and electrophysiology data were analyzed with Clampfit 10.2 (Molecular Devices) except where otherwise noted. The FIJI toolboxes MosiacJ^58^ and Simple Neurite Tracer^59^ were used for morphological reconstructions. Statistical analysis was performed in Prism software (GraphPad). Statistical significance was assessed with two-tailed *t*-tests, ANOVAs with post-hoc *t*-tests, or Mann-Whitney tests as indicated in the text or legends. Specific values *t*_df_, *F*_df_, *p*, and *r*^2^ values are reported in the figure legends with *r*^2^ calculated as *t*^2^/(*t*^*2*^ + df). In all figures, error bars indicate S.E.M.s, and statistical significance (*p* < 0.05, Bonferroni-corrected in the case of post-hoc tests following ANOVA) is denoted with a black line and star above the bar graph.

### Reporting summary

Further information on research design is available in the Nature Research Reporting Summary linked to this article.

## Supplementary information

Supplementary Information

Peer Review

Reporting Summary

Description of Additional Supplementary Files

Supplementary Data 1

Supplementary Data 2

## Data Availability

Full results of yeast two-hybrid and mass spectrometry analyses are provided in Supplementary Data 1 and 2. Other data that support the findings of this study are available from the corresponding author upon reasonable request.
