# Supplementary material for: Synaptotagmin 17 controls neurite outgrowth and synaptic physiology via distinct cellular pathways
Source: Nat Commun. 2019 Aug 6;10:3532. doi: 10.1038/s41467-019-11459-4 (PMC6684635; doi:10.1038/s41467-019-11459-4)
Supplement: Supplementary file 1 — Supplementary Information [file 41467_2019_11459_MOESM1_ESM.pdf]

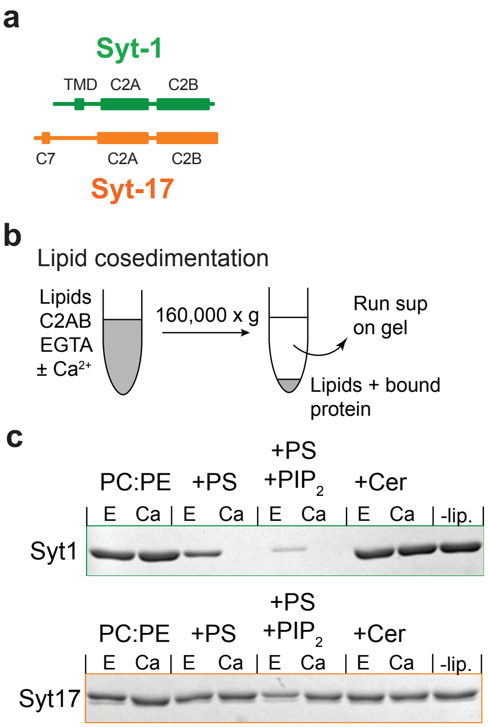

Supplementary Fig. 1
Syt-17 C2AB does not bind phospholipids. (a) Domain structure of syt-1 (green) and syt-17 (orange). “TMD” indicates the transmembrane domain of syt-1 and “C7” indicates the cysteine-rich N-terminal patch of syt-17. “C2A” and “C2B” denote the two C2 domains.
(b) Diagram of lipid cosedimentation experiment to determine Ca^{2+} dependent and independent lipid binding by the C2AB domains of syt-1 and syt-17. (c) Coomassie-stained SDS-PAGE gels showing residual protein in the supernatant (sup) following cosedimentation. Syt-1 C2AB bound phosphatidylserine (PS)-bearing vesicles in a Ca^{2+} dependent manner, and bound to vesicles that harbored phosphatidylinositol 4,5-bisphosphate (PIP_2), in both absence and presence of Ca^{2+}. Syt-17 C2AB did not bind lipids in either the presence or absence of Ca^{2+}. PE: phosphatidylethanolamine; PC: phosphatidylcholine; Cer: ceramide. *In vitro* experiments were performed with three independent preparations.

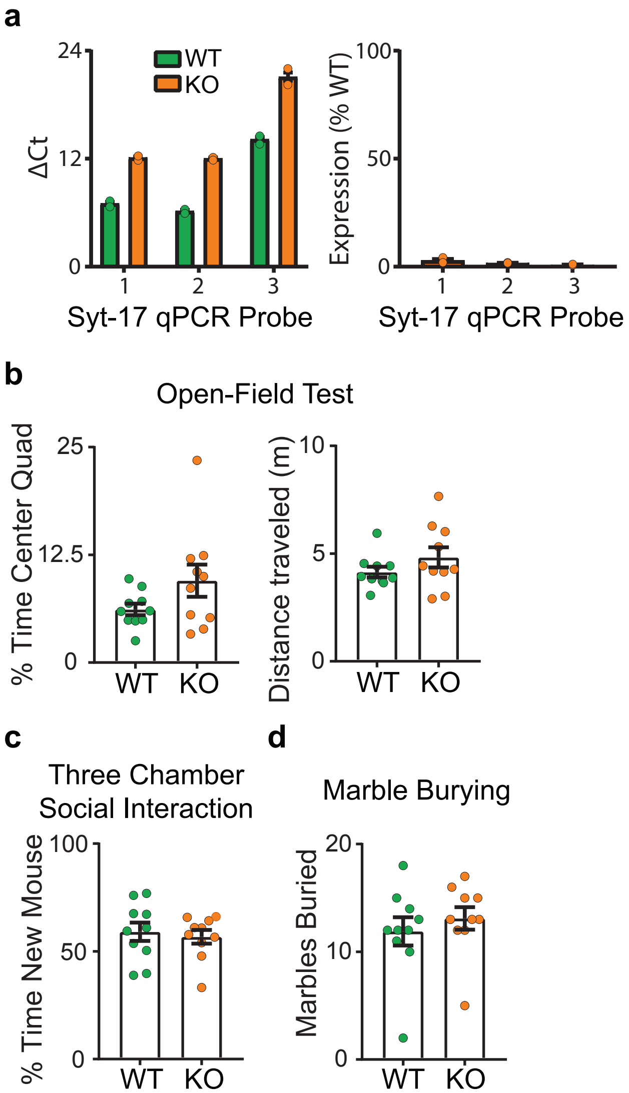

Supplementary Fig. 2

Validation and behavioral characterization of a syt-17 KO mouse model. (a) RT-PCR validation of the KO in brain tissue harvested from young (15 day postnatal) mice. Loss of transcript expression in the KO was validated with three distinct sets of RT-PCR primers (see methods). (b) Six-week-old mice of either gender, and from >3 breeding lines, were placed in an open field, and the time spent in the center quadrant (a metric inversely-related to anxiety; left) and total distance traveled (right) was quantified. No differences were observed between genotypes ($p>0.1$, two-sample t-test). (c) Animals were subject to a three-chamber social interaction test; relative preference for the novel stimulus mouse did not differ between genotypes ($p>0.1$, two-sample t-test). (d) Animals were place in a cage with a grid of marbles, and the number of marbles buried by the animal (a metric of perseveration) was quantified; no difference was observed between genotypes ($p>0.1$, two-sample t-test). All error bars indicate S.E.M.s.

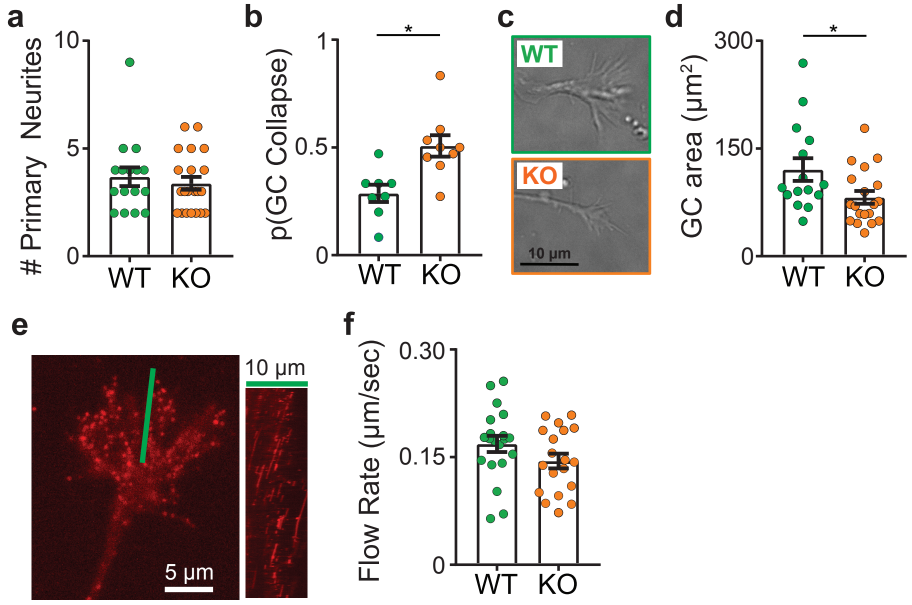

Supplementary Fig. 3

Abnormal growth cones despite typical neurite number and actin flow dynamics in syt-17 KO neurons. (a) The number of primary neurites per neuron at 2-3 DIV did not differ between WT and KO neurons ($p>0.1$ two-sample t-test). (b) The probability of spontaneous axonal growth cone collapse was higher in syt-17 KO ($t_{15}=3.382$, $p=0.004$, $r^2=0.433$, $mean_{wt}=0.29\pm0.04$ probability, $mean_{ko}=0.51\pm0.05$, $N_{wt}=8$ and $N_{ko}=9$ neurons). (c) 100x DIC images of axonal growth cones from WT and KO neurons. Scale bar indicates 10 µm. (d) Axonal growth cones were significantly smaller in neurons lacking syt-17 ($t_{32}=2.261$, $p=0.03$, $r^2=0.138$, $mean_{wt}=120.76\pm15.77$ µm^2, $mean_{ko}=81.95\pm8.86$, $N_{wt}=15$ and $N_{ko}=19$ neurons). (e) Neurons (2-4 DIV) were incubated with low concentrations of SiTMR-KrabC to label the barbed end of actin filaments, and subsequently imaged at 37°C in the presence of 5% CO_2. Scale bar (white) represents 5 µm. The kymograph on right corresponds to the indicated linescan (green). (f) The rate of actin retrograde flow did not differ between WT and KO neurons ($p>0.1$ two-sample t-test). All error bars indicate S.E.M.s.

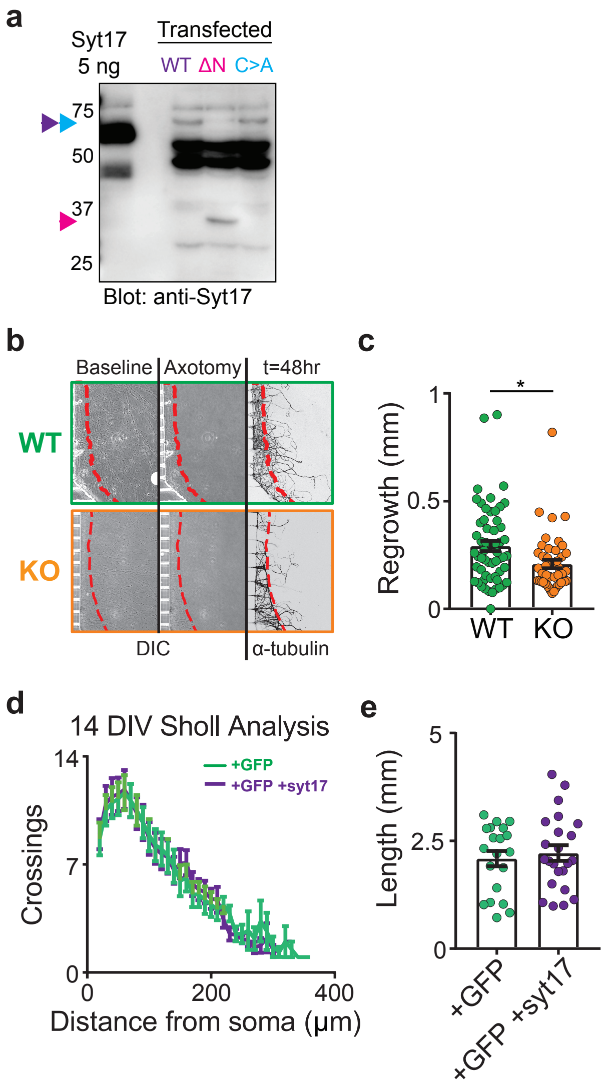

Supplementary Fig. 4

Overexpression of syt-17 mutants; KO of syt-17 impairs axonal regrowth after injury; syt-17 overexpression does not affect dendritic morphology. (a) Western blot showing equivalent overexpression of WT syt-17 and the delta-N and C>A mutants. Hippocampal neurons were infected with lentivirus expressing GFP and one of the three constructs, lysed at 14 DIV, and immunoblotted using an antibody we developed against the syt-17 C2B domain (Covance WI508). The positions of the syt-17 constructs are indicated with colored arrows. Note the large amount of non-specific binding that unfortunately precluded the use of this antibody for immunostaining. (b) WT and KO neurons were grown in microfluidic chambers, subject to axotomy at 14 DIV, then fixed 48 hrs later and immunostained for β-tubulin to assay regrowth. (c) Syt-17 KO neurons exhibited significantly less regrowth than WT neurons (t_{99}=2.56, p=0.012, r^2=0.062, $mean_{wt}$=0.29±0.02 μm, $mean_{ko}$=0.21±0.02, N_{wt}=57 and N_{ko}=44 axons). Regrowth measurements were made from two independent preparations of neurons. (d) Neurons were transfected with GFP with or without WT syt-17 at 3 DIV, fixed at 14 DIV, and their dendritic arbors were subject to Sholl analysis. No difference was observed between WT and overexpressing neurons either for branching complexity or (e) total dendrite length (p>0.1, two-sample t-test). Overexpression experiments were performed with at least three independent preparations of neurons. All error bars indicate S.E.M.s.

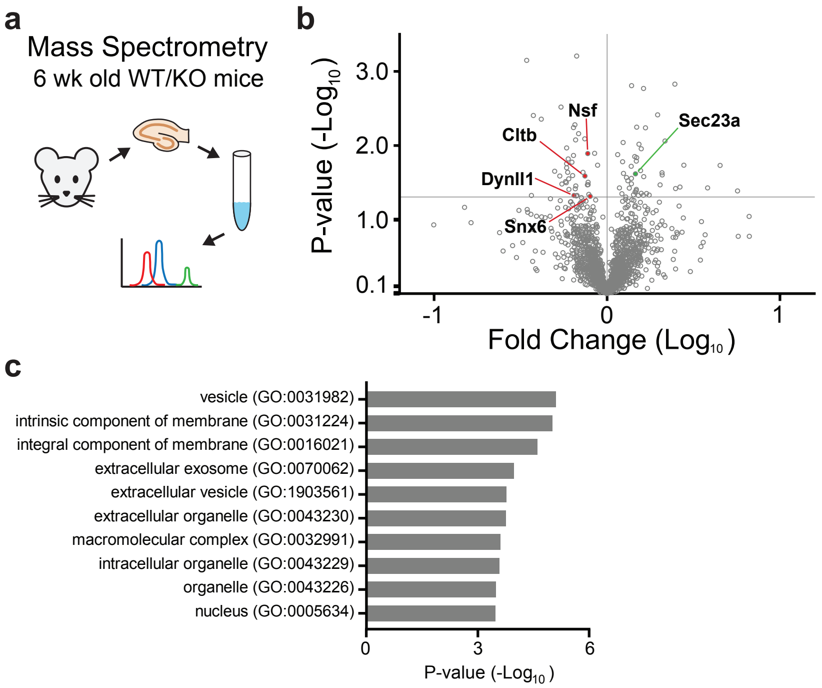

Supplementary Fig. 5

Multiplex mass-spectrometry proteomics to identify proteins differentially expressed in hippocampal tissue from adult syt-17 KO mice. (a) Hippocampi from adult syt-17 KO mice or WT littermates were harvested and lysed for proteomic analysis. (b) Volcano plot summarizing the analysis of differentially expressed proteins according to their P-value (y-axis) and their relative abundance ratio (log_2 fold change) between syt-17 WT and KO hippocampi. (c) Functional enrichment analysis of changed proteins by Fisher's exact test. Proteins found altered in syt-17 KO hippocampus are significantly enriched in GO terms related to vesicles and membrane components. Measurements were made from hippocampal tissue from 3 WT and 2 KO animals from separate breeding pairs. See also Supplementary Data 1.

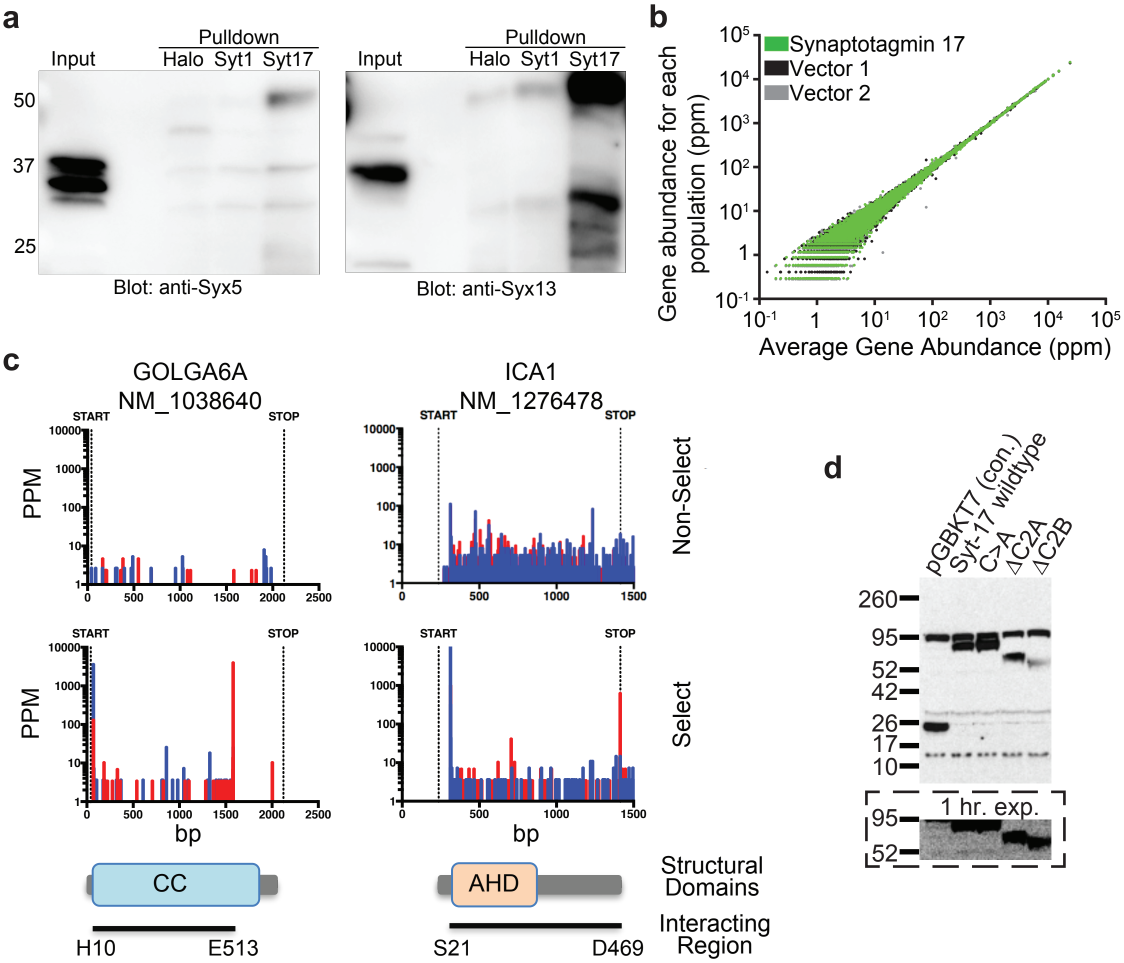

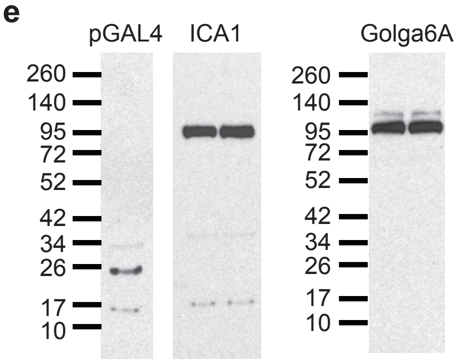

Supplementary Fig. 6
Identification and domain mapping of potential syt-17 interactors. (a) Immunoblots showing pulldown of syntaxin-5 or -13 by syt-17-Halo. Neither SNARE showed detectible binding to syt-17 C2AB. (b) A yeast two-hybrid screen was performed on the delta-C2A form of syt-17 to identify potential interactors. Plot shows the abundance of genes in the individual non-selected populations grown in the presence of histidine as a function of the average abundance across the three populations. (c) Top: The abundance of each fusion point between the indicated gene of interest and the Gal4-activation domain within the non-selected sub-population (top) and the selected population (bottom). Blue lines depict the position of the 5' end of prey gene fragments, red lines depict 3' ends of the prey gene fragments. Bottom: Schematic of corresponding candidate interacting proteins indicating the interacting region and structural domains, including the coiled-coil domain (CC) of GOLGA6A and the arfaptin homology domain (AHD) of ICA1. See also Supplementary Data 2. (d) Immunoblot (anti-myc) of Gal4-DNA binding domain fusions of pGBKT7 (vector alone control) or syt-17 constructs. Mutants include syt-17 with N-terminal cysteines mutated to alanines (C>A), and deletions of either the C2A (delta-C2A) or C2B (delta-C2B) domains. (e) Immunoblot (anti-HA) of Gal4-activation domain fusions of pGAL4 (vector alone pGAL4-AD), Golga6A or ICA1.

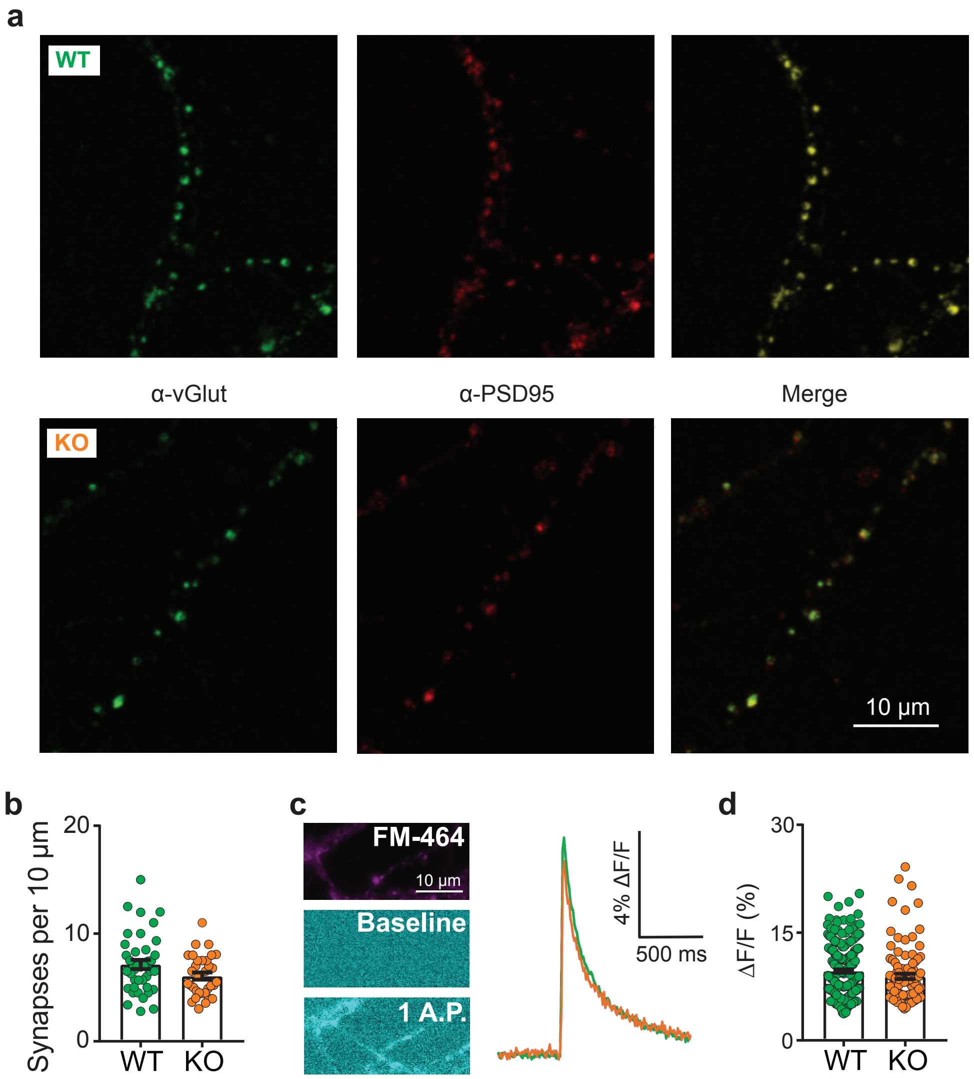

Supplementary Fig. 7

Normal synapse density and presynaptic Ca^{2+} influx in syt-17 KO neurons. (a) Neurons (14 DIV) were fixed and immunostained for vGlut and PSD-95. Scale bar indicates 10 µm. (b) The number of synapses (defined as puncta positive for both pre- and postsynaptic markers) per unit dendrite did not significantly differ between groups (though a non-significant trend towards fewer synapses in the KO was noted, p=0.06, two-sample t-test). (c) Presynaptic boutons were labelled by loading with FM-464 dye (left, top). Following loading with Fluo-5F AM Ca^{2+} dye, single action potentials were evoked with a field stimulation chamber (left, middle/bottom). Average Fluo-5F fluorescence change (deltaF/F) was quantified from individual boutons (right). (d) No significant difference in peak response was observed between genotypes (p=0.09, two-sample t-test). All error bars indicate S.E.M.s.

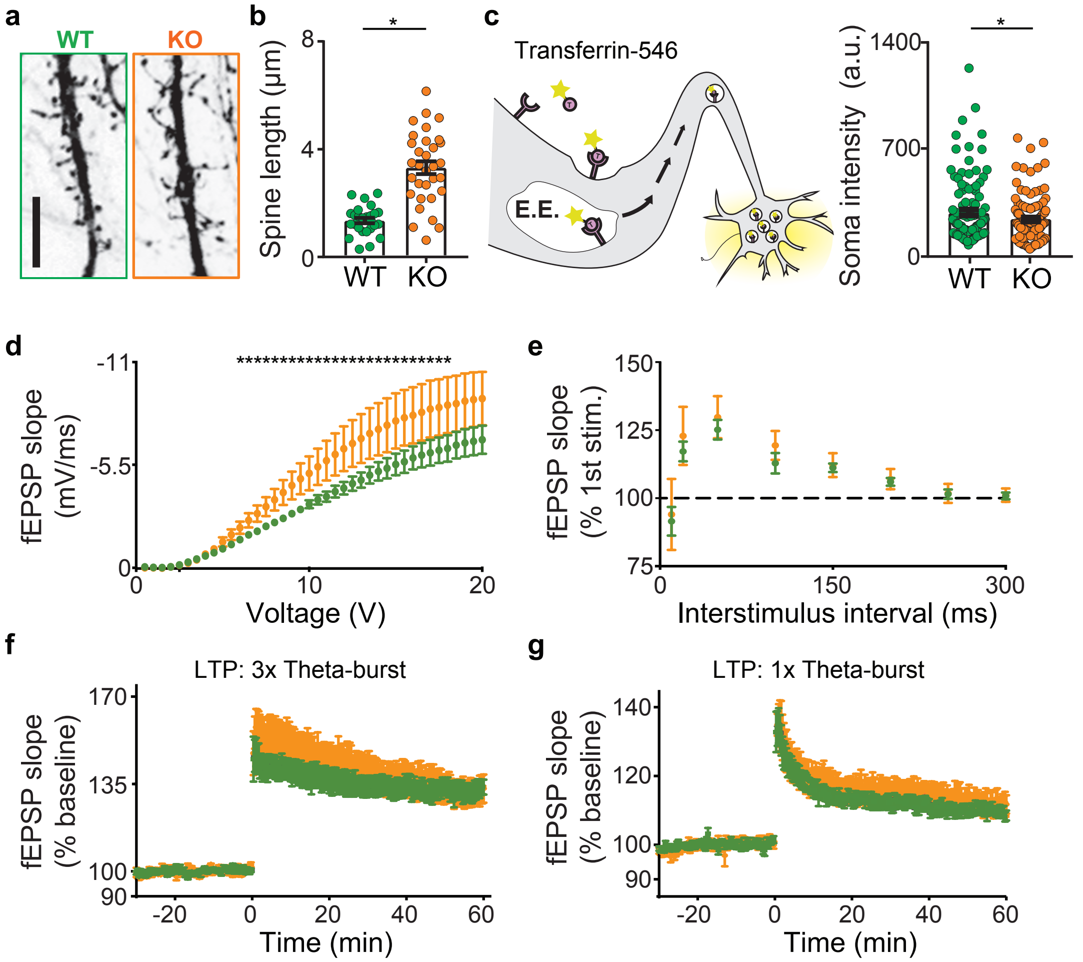

Supplementary Fig. 8

Abnormal dendritic spine morphology and constitutive endocytosis in syt-17 KO neurons. (a) Neurons were transfected at 3 DIV with soluble GFP and fixed at 14 DIV to visualize neuronal morphology. The dendritic spines of neurons lacking syt-17 more closely resemble filopodia than mature mushroom-type spines. (b) The average length of dendritic spines was significantly longer in syt-17 KO neurons (t_{57}=7.152, p<0.001, r^2=0.473, $mean_{wt}$=1.35±0.1 µm, $mean_{ko}$=3.31±0.24, N_{wt}=27 and N_{ko}=32 neurons). (c) Left: Transferrin, tagged with Alexa 546, is internalized via clathrin-mediated endocytosis, routed to early endosomes in a Rab-5 dependent manner, and trafficked to the soma. Right: Average somatic Transferrin-546 intensity post-incubation is reduced in syt-17 KO (t_{220}=1.898, p=0.05, r^2=0.016, $mean_{wt}$=292.33±20.3 a.u., $mean_{ko}$=246.05±13.9, N_{wt}=108 and N_{ko}=114 neurons), demonstrating impaired endocytic recycling. All measurements were made from at least three independent preparations of neurons. (d) Input-output curves of field excitatory postsynaptic potentials (fEPSPs) measured along the Shaffer collaterals from hippocampal slices of WT and KO animals. Synaptic responses are significantly elevated in the KO along a range of stimulation intensities. (e) Paired-pulse facilitation in the same slices over a range of interstimulus intervals. No differences between genotypes were observed. (f) Long-term potentiation (LTP) along the Shaffer collateral pathway in acute hippocampal slices, induced with three theta-burst stimulations; no significant difference between WT and KO slices was observed (t_{15}=0.008, p=0.99, $mean_{wt}$=31.64±3.06 % of baseline, $mean_{ko}$=31.58±5.6, N_{wt}=8 slices from three mice, N_{ko}=9 slices from four mice). (g) LTP induced with a weaker stimulation paradigm (one theta-burst) similarly produced no difference between WT and KO (t_{14}=-0.26, p=0.92, $mean_{wt}$=9.75±1.38 % of baseline, $mean_{ko}$=10.58±2.66, N_{wt}=8 slices from two mice, N_{ko}=8 slices from four mice). All error bars indicate S.E.M.s.
