# Supplementary material for: Synaptotagmin 17 controls neurite outgrowth and synaptic physiology via distinct cellular pathways
Source: Nat Commun. 2019 Aug 6;10:3532. doi: 10.1038/s41467-019-11459-4 (PMC6684635; doi:10.1038/s41467-019-11459-4)
Supplement: Supplementary file 3 — Reporting Summary [file 41467_2019_11459_MOESM3_ESM.pdf]

nature research

Corresponding author(s): *Double-blind peer review submissions: write DBPR and your manuscript number here instead of author names.*

Last updated by author(s): Jul 6, 2019

Reporting Summary

Nature Research wishes to improve the reproducibility of the work that we publish. This form provides structure for consistency and transparency in reporting. For further information on Nature Research policies, see Authors & Referees and the Editorial Policy Checklist.

Statistics

For all statistical analyses, confirm that the following items are present in the figure legend, table legend, main text, or Methods section.

n/a	Confirmed	
☐	☒	The exact sample size (n) for each experimental group/condition, given as a discrete number and unit of measurement
☐	☒	A statement on whether measurements were taken from distinct samples or whether the same sample was measured repeatedly
☐	☒	The statistical test(s) used AND whether they are one- or two-sided<br>*Only common tests should be described solely by name; describe more complex techniques in the Methods section.*
☒	☐	A description of all covariates tested
☐	☒	A description of any assumptions or corrections, such as tests of normality and adjustment for multiple comparisons
☐	☒	A full description of the statistical parameters including central tendency (e.g. means) or other basic estimates (e.g. regression coefficient) AND variation (e.g. standard deviation) or associated estimates of uncertainty (e.g. confidence intervals)
☐	☒	For null hypothesis testing, the test statistic (e.g. F, t, r) with confidence intervals, effect sizes, degrees of freedom and P value noted<br>*Give P values as exact values whenever suitable.*
☒	☐	For Bayesian analysis, information on the choice of priors and Markov chain Monte Carlo settings
☒	☐	For hierarchical and complex designs, identification of the appropriate level for tests and full reporting of outcomes
☐	☒	Estimates of effect sizes (e.g. Cohen's d, Pearson's r), indicating how they were calculated

Our web collection on statistics for biologists contains articles on many of the points above.

Software and code

Policy information about availability of computer code

Data collection	n/a
Data analysis	n/a

For manuscripts utilizing custom algorithms or software that are central to the research but not yet described in published literature, software must be made available to editors/reviewers. We strongly encourage code deposition in a community repository (e.g. GitHub). See the Nature Research guidelines for submitting code & software for further information.

Data

Policy information about availability of data

All manuscripts must include a data availability statement. This statement should provide the following information, where applicable:

- Accession codes, unique identifiers, or web links for publicly available datasets
- A list of figures that have associated raw data
- A description of any restrictions on data availability

Full results of yeast two-hybrid and mass spectrometry analyses are provided in Supplementary Data 1 and 2. Other data that support the findings of this study are available from the corresponding author upon reasonable request.

Field-specific reporting

Please select the one below that is the best fit for your research. If you are not sure, read the appropriate sections before making your selection.

☒ Life sciences ☐ Behavioural & social sciences ☐ Ecological, evolutionary & environmental sciences

Life sciences study design

All studies must disclose on these points even when the disclosure is negative.

Sample size	Sample sizes were comparable or higher to those typical in the field. All experiments were replicated with at least three independent preparations.
Data exclusions	Data were only excluded in the case of experimental failure (ex., if a construct failed to express, that entire preparation was excluded), and never purely to remove outliers or otherwise "clean" data.
Replication	All experiments are described in detail for future replication. The two main phenotypic effects were replicated within our lab: the outgrowth phenotype was observed independently by three experimenters, and the increase in EPSC amplitude was observed independently by two.
Randomization	Cells were generally selected randomly for experiments (ex., by arbitrarily moving a field of view) except as reported in the methods. For transfections, multiple coverslips from the same animals were transfected with various constructs; the selection for this was random.
Blinding	Investigators were blinded whenever feasible (especially for experiments that required extensive manual intervention).

Reporting for specific materials, systems and methods

We require information from authors about some types of materials, experimental systems and methods used in many studies. Here, indicate whether each material, system or method listed is relevant to your study. If you are not sure if a list item applies to your research, read the appropriate section before selecting a response.

Materials & experimental systems

n/a	Involved in the study	
☐	☒	Antibodies
☐	☒	Eukaryotic cell lines
☒	☐	Palaeontology
☐	☒	Animals and other organisms
☒	☐	Human research participants
☒	☐	Clinical data

Methods

n/a	Involved in the study	
☒	☐	ChIP-seq
☒	☐	Flow cytometry
☒	☐	MRI-based neuroimaging

Antibodies

Antibodies used	Anti-PSD95 Abcam Ab18258<br>Anti-vGlut Millipore Ab5905<br>Anti-GFP Abcam Ab13970<br>Anti-syt-17 Covance WI508
Validation	Validation data for the Covance anti-syt17 is available in the Supplemental Figures. Validation data for the remaining antibodies is available on the manufacturers' websites.

Eukaryotic cell lines

Policy information about cell lines

Cell line source(s)	HEK293t cells (Sigma) were used to manufacture lentivirus and for some initial testing of constructs
Authentication	The line was obtained directly from the manufacturer, no additional authentication was performed.
Mycoplasma contamination	The line was obtained directly from the manufacturer, no additional testing was performed.
Commonly misidentified lines (See ICLAC register)	n/a

Animals and other organisms

Policy information about studies involving animals; ARRIVE guidelines recommended for reporting animal research

Laboratory animals	Male or female C57B6J syt-17 knockout or wildtype mice, age 0-6 months

Wild animals	n/a
Field-collected samples	n/a
Ethics oversight	All animal experiments were conducted at the University of Wisconsin-Madison, and protocols were reviewed and approved by the university's Animal Care and Use Committee (assurance # A3368-01). The All relevant ethics regulations for animal testing and research were followed, and the guidelines set by the NIH Guide for the Care and Use of Laboratory Animals handbook were conformed to in all cases.

Note that full information on the approval of the study protocol must also be provided in the manuscript.
