# Supplementary material for: Synaptotagmin 17 controls neurite outgrowth and synaptic physiology via distinct cellular pathways
Source: Nat Commun. 2019 Aug 6;10:3532. doi: 10.1038/s41467-019-11459-4 (PMC6684635; doi:10.1038/s41467-019-11459-4)
Supplement: Supplementary file 4 — Description of Additional Supplementary Files [file 41467_2019_11459_MOESM4_ESM.docx]

**Description of Additional Supplementary Files**

File Name: Supplementary Data 1
Description: Mass spectrometry of WT/KO hippocampal lysates.

File Name: Supplementary Data 2
Description: Results of a DEEPN yeast two-hybrid screen against syt-17 ∆C2A.
