# Supplementary material for: Synaptotagmin 17 controls neurite outgrowth and synaptic physiology via distinct cellular pathways
Source: Nat Commun. 2019 Aug 6;10:3532. doi: 10.1038/s41467-019-11459-4 (PMC6684635; doi:10.1038/s41467-019-11459-4)
Supplement: Supplementary file 2 — Peer Review [file 41467_2019_11459_MOESM2_ESM.pdf]

Reviewers' comments:

Reviewer #1 (Remarks to the Author):

Synaptotagmin (Syt) proteins are important regulators of exocytosis with e.g. Syt1or Syt2 being Ca2+ sensors for triggering neurotransmitter release. Ruhl et al investigated the function of Syt17 in hippocampal neurons, using a wide spectrum of methods. Syt17 is structurally different from other Syts and its function is largely elusive. The authors now report that Syt17 has two distinct functions in hippocampal neurons, associated with two distinct pools of Syt17. They find one pool of Syt17 to localize to the somatic Golgi, where it functions in vesicle trafficking from ER to Golgi and supports neurite growth and regrowth after injury. The second pool localizes to neurites and is involved in regulating synaptic strength and LTD by being engaged in AMPA receptor recycling. These findings are novel and very interesting. They shed light on the previously elusive function of Syt17. Overall the manuscript is concise and clear. I only have a few comments of which I hope that they help further improving this very good manuscript.

Major
1) LTP in addition to LTD
The authors show that in Syt17 KO synaptic strength is increased due to increased number of postsynaptic AMPA receptors while LTD is impaired. Postsynaptic Syt1 and Syt7 were recently shown to mediate Ca2+ -dependent exocytosis of AMPA receptors during NMDA -induced LTP in CA1 (Wu et al., Nature, 2017). Hence, it would be interesting to see, if this form of LTP is altered in Syt17 KO or if synaptic strength is already “saturated” due to the increased abundance of AMPA receptors in the KO and cannot be increased further by LTP.

2) Ca imaging
Fluo-5F might be quite dark under resting conditions, which can make $\Delta F/F0$ normalization problematic. Hence, either a proof should be provided that F0 is well above 0 or else a normalization to the FM-464 fluorescence could be used.

3) Extend figure legends
The information given about number of animals, number of cells and number of measurements and what individual data points show is very scarce in the figure legends and not included in the main text. Also the mean and SEM are not stated. This information should be enhanced in the figure legends or the main text, rather than just giving df values.

4) Statistics
If I understood correctly, the authors used a t-test throughout. However, justification (e.g. normal distribution, equal variance etc.) for this is not explained in the methods. Some data (e.g. Fig. 2d and Fig. 3c) cover more than two groups and would require an ANOVA or ANOVA on ranks to avoid multiple testing.

Minor
Abstract line 35 should read “The function …”

Page 4, line 105: “data not shown”. I think this does not conform to journal style and data could be shown in the supplement.

Page 6, line 150: Should read “pyramidal neurons …”

Fig. 3a: “purple” is blue in my printout.

Reviewer #2 (Remarks to the Author):

The manuscript by Ruhl et al. (NCOMMS-19-00844) examined the role of Syt-17, a neglected isoform of a large family of synaptotagmins. Combined with cell biology, biochemical, and synaptic physiological analysis in cultured neurons and brain slices of syt-17 KO mice, the authors reported a dual-function of this protein in coordinating the import of early secretory vesicle trafficking from the ER to the Golgi, supporting neurite outgrowth, and in mediating endocytic recycling of AMPARs, modulating synaptic strength and plasticity.

Overall, this is an interesting study and KO phenotypes are intriguing. The manuscript is logically presented and most of the data are solid and generally high quality. It represents a great deal of work, as evidenced by comprehensive and quantitative analysis of multiple approaches. However, I have two main concerns and several specific comments; addressing these would be necessary to strengthen their study and thus make an important contribution to the field.

Main Concerns:

1. The proposed role of syt-17 in regulating early secretory trafficking by forming a “cargo import complex” with Golgi proteins GOLGA6A and ICA1 is not functionally validated. Knocking down syt-17-binding partners or expressing their binding-defective mutants would be necessary to establish the causal role of this protein complex in mediating neurite growth and regeneration after injury. The authors might also examine whether knocking down GOLGA6A or ICA1 impairs early secretory trafficking from the ER to Golgi. These experiments will provide new mechanistic insights into syt-17-mediated coordination of early secretory vesicle trafficking and neurite outgrowth.

2. While synaptic phenotypes of syt-17 KO mice are intriguing, the mechanism underlying the syt-7-mediated internalization and endocytic recycling of AMPARs was not explored. In addition, co-staining of Rab5 with AMPARs and syt-17 in dendritic spines in WT or syt-17 overexpressed neurons will support the idea of syt-17-mediated endocytic recycling of AMPARs. Addressing this will substantially advance the field.

Specific Comments:

3. Fig1d-f: Syt-17 localizes to the Golgi, and early endosomes in hippocampal neurons. The targeting or distribution data need to be confirmed by immunostaining of endogenous proteins of various organelle markers, including early and late endosomes, recycling endosomes, mitochondria, ER, and Golgi. Overexpressed proteins will likely cause artificial targeting due to oversaturation of sorting mechanisms. For example, in Fig1d, pHluorin-syt-17 in neurites appears as tubular structures, which resemble mitochondria rather than endosomal structures.

4. Fig3c: Expressing either of two mutants abolished the overexpression phenotype: these data are less informative as both mutant constructs are unlikely to have a dominant-negative effect. A new study showing failed rescue of the KO neuron phenotype with these mutants would support their conclusion.

5. Fig3e-3f: Enhanced regrowth capacity after injury is rather subtle. Even KO neurons display some degree of regeneration (FigS4b-c). Thus, rescue of KO neuron regeneration by expressing syt-17 is necessary.

6. FigS4d-e: While KO neurons display reduced growth in both axons and dendrites, the overexpression phenotype is specific to axons. This is an interesting observation, which might indicate a unique nature of syt-17-mediated secretary trafficking in axons vs dendrites. A high-resolution analysis of secretary cargos trafficking in axons vs dendrites might provide insights into this issue.

7. In the experiments where multiple comparisons are made, t-tests were used to compute statistics rather than ANOVA tests with correction for multiple comparisons.

Reviewer #3 (Remarks to the Author):

In this study, the authors investigate the functional of role of synaptotagmin-17 (also known as B/K protein) in neurons. Synaptotagmin-17 binds to golgin as well as GOLGA6A, and is involved in the function of Golgi and early endosomes. The authors propose that synaptotagmin-17 is thus a quite an unusual synaptotagmin isoform. Overall, this study contains important information on the function of this protein and the experiments are conducted quite well. However, I think there are two issues that need to be addressed in the text prior to publication.

1. From the data presented it is unclear if synaptotagmin-17 is indeed a synaptotagmin isoform. I think that the definition of a "synaptotagmin" is a protein with N-terminal transmembrane region and C-terminal tandem C2 domains. B/K protein, or Synaptotagmin-17, is lacking N-terminal transmembrane region. If the authors extended definition of synaptotagmins apply, then every Doc2 proteins should be called a synaptotagmin isoform. Therefore, in my opinion, B/K protein (Synaptotagmin-17) is not a synaptotagmin isoform and accordingly it displays unusual characteristics from those of synaptotagmins. This point needs further justification by the authors.
2. I think knocking out B/K protein is very specific, but as B/K protein interacts with important Golgi and early endosome proteins, it is likely involved in the functions of the Golgi as well as endosomes. As the Golgi is essential for several cellular functions, impairing the Golgi function can produce many different types of physiological changes including those the manuscript described. These potential indirect effects of B/K or synaptotagmin-17 should be acknowledged.

HHMI
HOWARD HUGHES MEDICAL INSTITUTE

Point-by-point response to concerns raised by the referees

Reviewer #1:

Major
1) LTP in addition to LTD
The authors show that in Syt17 KO synaptic strength is increased due to increased number of postsynaptic AMPA receptors while LTD is impaired. Postsynaptic Syt1 and Syt7 were recently shown to mediate Ca2+ -dependent exocytosis of AMPA receptors during NMDA -induced LTP in CA1 (Wu et al., Nature, 2017). Hence, it would be interesting to see, if this form of LTP is altered in Syt17 KO or if synaptic strength is already "saturated" due to the increased abundance of AMPA receptors in the KO and cannot be increased further by LTP.

Please see the following long-term potentiation measurements, conducted in the Shaffer collateral pathway of acute hippocampal slices from 6-10 week-old mice. WT data points are shown in green, KO in orange:

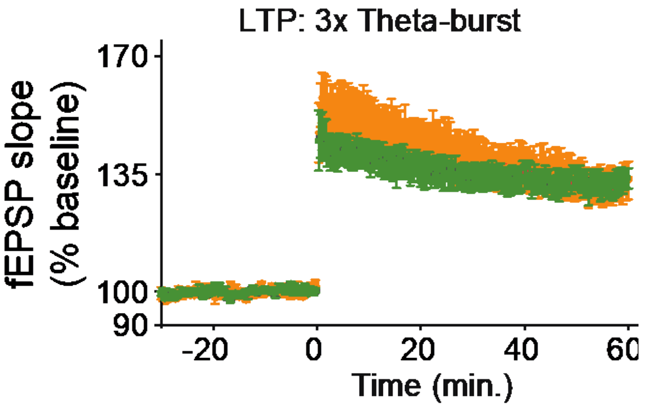

Using this induction paradigm (three theta-bursts), we find no significant difference between WT and KO slices (t_{15}=0.008, p=0.99, $mean_{wt}$=31.64±3.06, $mean_{ko}$=31.58±5.6, N_{wt}=8 slices from three mice, N_{ko}=9 slices from four mice).

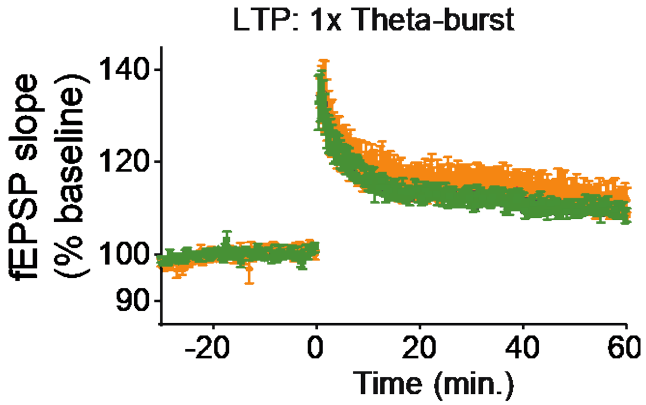

We also conducted an independent set of LTP experiments using a weaker induction paradigm (one theta burst), to ensure that either increased or decreased potentiation in the KO would be detectible. Similar to the experiments with strong stimulation, no alteration in LTP was observable under this paradigm (t_{14}=-0.26, p=0.92, $mean_{wt}$=9.75±1.38, $mean_{ko}$=10.58±2.66, N_{wt}=8 slices from two mice, N_{ko}=8 slices from four mice).

University of Wisconsin, Department of Neuroscience, WIMR2 1111 Highland Ave. (room 5505-mail; 9557-office), Madison, Wisconsin 53706
Tel. (608) 263-1762 • Fax. (608) 265-7821 • e-mail: chapman@wisc.edu
http://www.hhmi.org/research/investigators/chapman.html
http://neuro.wisc.edu/faculty/chapman.asp
http://www.neuro.wisc.edu/chapman/Chapman_Lab/Home.html

Collectively, these experiments demonstrate the syt-17 KO animals have no deficit in LTP, but that their defect in synaptic plasticity is specific to LTD. These results are consistent with syt-17 playing a key role in AMPAR endocytosis (LTD), but not AMPAR exocytosis (LTP). Syt-17 is expressed on Rab5-positive early endosomes, which play a crucial function in LTD (Brown et al., 2005; Hirling, 2009), but do not appear to be directly involved in LTP or AMPAR exocytosis, which are unaffected by downregulation of Rab5 (Brown et al., 2007; Bowen et al., 2017). The null effect of syt17 KO on LTP thus strengthens the case for a specific role of syt-17 in AMPAR internalization.

These results have been added to Supplementary Figure 8; the text and legends have been modified accordingly.

2) Ca imaging

Fluo-5F might be quite dark under resting conditions, which can make ΔF/F0 normalization problematic. Hence, either a proof should be provided that F0 is well above 0 or else a normalization to the FM-464 fluorescence could be used.

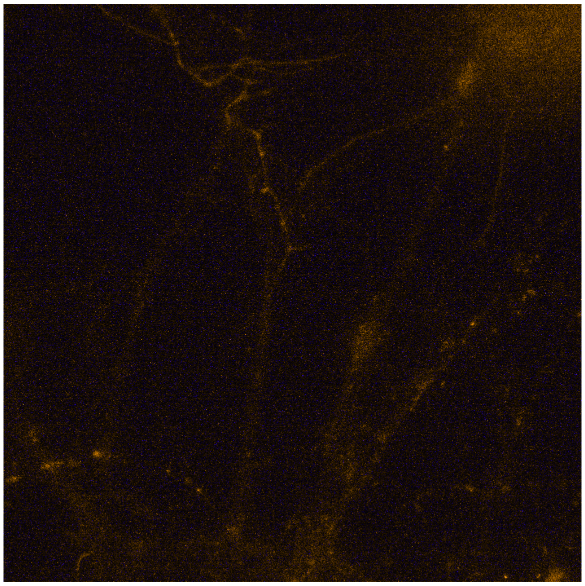

This is an important and oft-overlooked point, we thank the reviewer for raising it. A representative raw imaging field of view at baseline is shown to the right. In our configuration, resting Fluo-5F fluorescence is detectable above baseline, sufficient to identify neuronal processes, and exclude most glia (based on their high baseline fluorescence). To quantify this, we compared the resting fluorescence of synaptic ROIs to nearby non-neuronal ROIs (i.e., background areas of the coverslip with no cells). The graph below is a histogram in which the x-axis represents the statistical significance (p value) comparing synaptic ROIs to background (presented on a log scale for clarity) for each field of view. In every field of view imaged in our study, baseline fluorescence in synaptic ROIs was significantly higher than background.

University of Wisconsin, Department of Neuroscience, WIMR2 1111 Highland Ave. (room 5505-mail; 9557-office), Madison, Wisconsin 53706
Tel. (608) 263-1762 • Fax. (608) 265-7821 • e-mail: chapman@wisc.edu
http://www.hhmi.org/research/investigators/chapman.html
http://neuro.wisc.edu/faculty/chapman.asp
http://www.neuro.wisc.edu/chapman/Chapman_Lab/Home.html

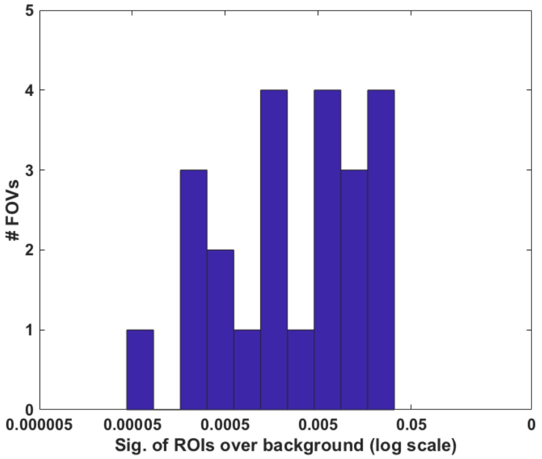

Further, we checked to ensure that this baseline fluorescence did not differ between WT and KO samples, which could potentially complicate interpretation of our ΔF/F0 values. WT synaptic ROIs exhibited a baseline fluorescence 11.65 +/- 2.1% higher than background, and KO synaptic ROIs exhibited a baseline fluorescence 11.43 +/- 1.24% higher than background (p=0.9).

This improves our confidence in our ΔF/F0 measurements, and we have revised our methods section to reflect these additional controls.

3) Extend figure legends
The information given about number of animals, number of cells and number of measurements and what individual data points show is very scarce in the figure legends and not included in the main text. Also the mean and SEM are not stated. This information should be enhanced in the figure legends or the main text, rather than just giving df values.

This is a good point; this information has been added to the figure legends.

4) Statistics
If I understood correctly, the authors used a t-test throughout. However, justification (e.g. normal distribution, equal variance etc.) for this is not explained in the methods. Some data (e.g. Fig. 2d and Fig. 3c) cover more than two groups and would require an ANOVA or ANOVA on ranks to avoid multiple testing.

We thank the reviewer for pointing this out; this issue has been corrected.

Minor
Abstract line 35 should read “The function …”

This has been corrected.

Page 4, line 105: “data not shown”. I think this does not conform to journal style and data could be shown in the supplement.

This has been removed.

University of Wisconsin, Department of Neuroscience, WIMR2 1111 Highland Ave. (room 5505-mail; 9557-office), Madison, Wisconsin 53706
Tel. (608) 263-1762 • Fax. (608) 265-7821 • e-mail: chapman@wisc.edu
http://www.hhmi.org/research/investigators/chapman.html
http://neuro.wisc.edu/faculty/chapman.asp
http://www.neuro.wisc.edu/chapman/Chapman_Lab/Home.html

Page 6, line 150: Should read "pyramidal neurons …"

This has been corrected.

Fig. 3a: "purple" is blue in my printout.

Strange, in ours it appeared purple. But we thank the referee for this feedback - we lightened the shade of purple for this condition, and will take care to consult with the editor on appropriate changes in the color scheme prior to publication if our study is accepted.

Reviewer #2:

Main Concerns:

1. The proposed role of syt-17 in regulating early secretory trafficking by forming a "cargo import complex" with Golgi proteins GOLGA6A and ICA1 is not functionally validated. Knocking down syt-17-binding partners or expressing their binding-defective mutants would be necessary to establish the causal role of this protein complex in mediating neurite growth and regeneration after injury. The authors might also examine whether knocking down GOLGA6A or ICA1 impairs early secretory trafficking from the ER to Golgi. These experiments will provide new mechanistic insights into syt-17-mediated coordination of early secretory vesicle trafficking and neurite outgrowth.

We thank the reviewer for raising this issue. The reviewer suggested two possible approaches to address this concern: knockdown of syt-17 binding partners, or expression of binding-defective mutants of syt-17. We have opted for the latter of these two options, as loss-of-function experiments with syt-17 binding partners are either previously published or beyond the scope of the present study. Regarding ICA1, it has been established that loss of this protein (aka ICA69) results in defects in secretory trafficking from the ER to the Golgi (Buffa et al., 2008), similar to our results with syt-17. In the case of GOLGA6A, the near-complete lack of knowledge about this protein (at the biochemical or functional levels) and lack of available reagents (validated antibodies, shRNAs, or tagged proteins) makes characterization of the role of GOLGA6A in secretory function and neural development a major undertaking, one that would close-to double the length of the present manuscript. To our mind, a detailed characterization of GOLGA6A and its interactions with syt-17 is a natural follow-up to our study, but is beyond the scope of the present work: characterizing the function of syt-17 in neurons.

University of Wisconsin, Department of Neuroscience, WIMR2 1111 Highland Ave. (room 5505-mail; 9557-office), Madison, Wisconsin 53706
Tel. (608) 263-1762 • Fax. (608) 265-7821 • e-mail: chapman@wisc.edu
http://www.hhmi.org/research/investigators/chapman.html
http://neuro.wisc.edu/faculty/chapman.asp
http://www.neuro.wisc.edu/chapman/Chapman_Lab/Home.html

However, we do see the argument that the postulated cargo import complex could be better functionally validated by expressing the binding-defective mutants. To that end, we have expanded our structure-function analysis of syt-17:

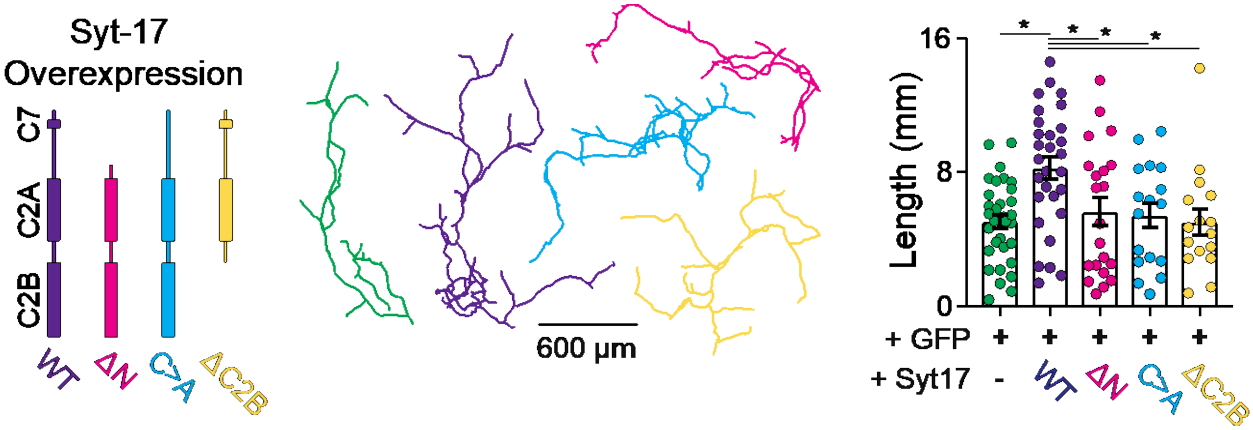

As is evident, the syt-17 C>A mutant that fails to bind ICA1 also fails to drive axonal outgrowth. Similarly, we have now included the syt-17 mutant which fails to bind GOLGA6A (C2B-deletion), and found that this mutant also fails to drive axonal outgrowth. Thus, mutations that disrupt the interaction of syt-17 with either of these Golgi-resident proteins disrupt the ability of the protein to enhance axonal outgrowth. This data has been incorporated into a revised Figure 3.

2. While synaptic phenotypes of syt-17 KO mice are intriguing, the mechanism underlying the syt-7-mediated internalization and endocytic recycling of AMPARs was not explored. In addition, co-staining of Rab5 with AMPARs and syt-17 in dendritic spines in WT or syt-17 overexpressed neurons will support the idea of syt-17-mediated endocytic recycling of AMPARs. Addressing this will substantially advance the field.

A representative image of the requested co-staining of Rab5/AMPARs/syt-17 is presented to the right. The traces represent normalized fluorescence in the indicated three micron linescan, traversing two dendritic spines of a 14DIV neuron. This has been added to the manuscript as Figure 7A. We think it makes a natural transition to the final portion of the paper, and appreciate the reviewer's suggestion. Additionally, we make reference in the text to previous studies demonstrating that dendritic rab5-positive endosomes colocalize with AMPARs.

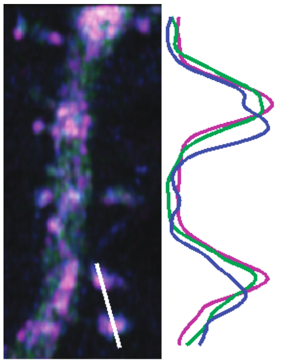

Specific Comments:

3. Fig1d-f: Syt-17 localizes to the Golgi, and early endosomes in hippocampal neurons. The targeting or distribution data need to be confirmed by

University of Wisconsin, Department of Neuroscience, WIMR2 1111 Highland Ave. (room 5505-mail; 9557-office), Madison, Wisconsin 53706
Tel. (608) 263-1762 • Fax. (608) 265-7821 • e-mail: chapman@wisc.edu
http://www.hhmi.org/research/investigators/chapman.html
http://neuro.wisc.edu/faculty/chapman.asp
http://www.neuro.wisc.edu/chapman/Chapman_Lab/Home.html

immunostaining of endogenous proteins of various organelle markers, including early and late endosomes, recycling endosomes, mitochondria, ER, and Golgi. Overexpressed proteins will likely cause artificial targeting due to oversaturation of sorting mechanisms. For example, in Fig1d, pHluorin-syt-17 in neurites appears as tubular structures, which resemble mitochondria rather than endosomal structures.

Please see below dual-color imaging of syt-17 with a mitochondrial stain and other markers.

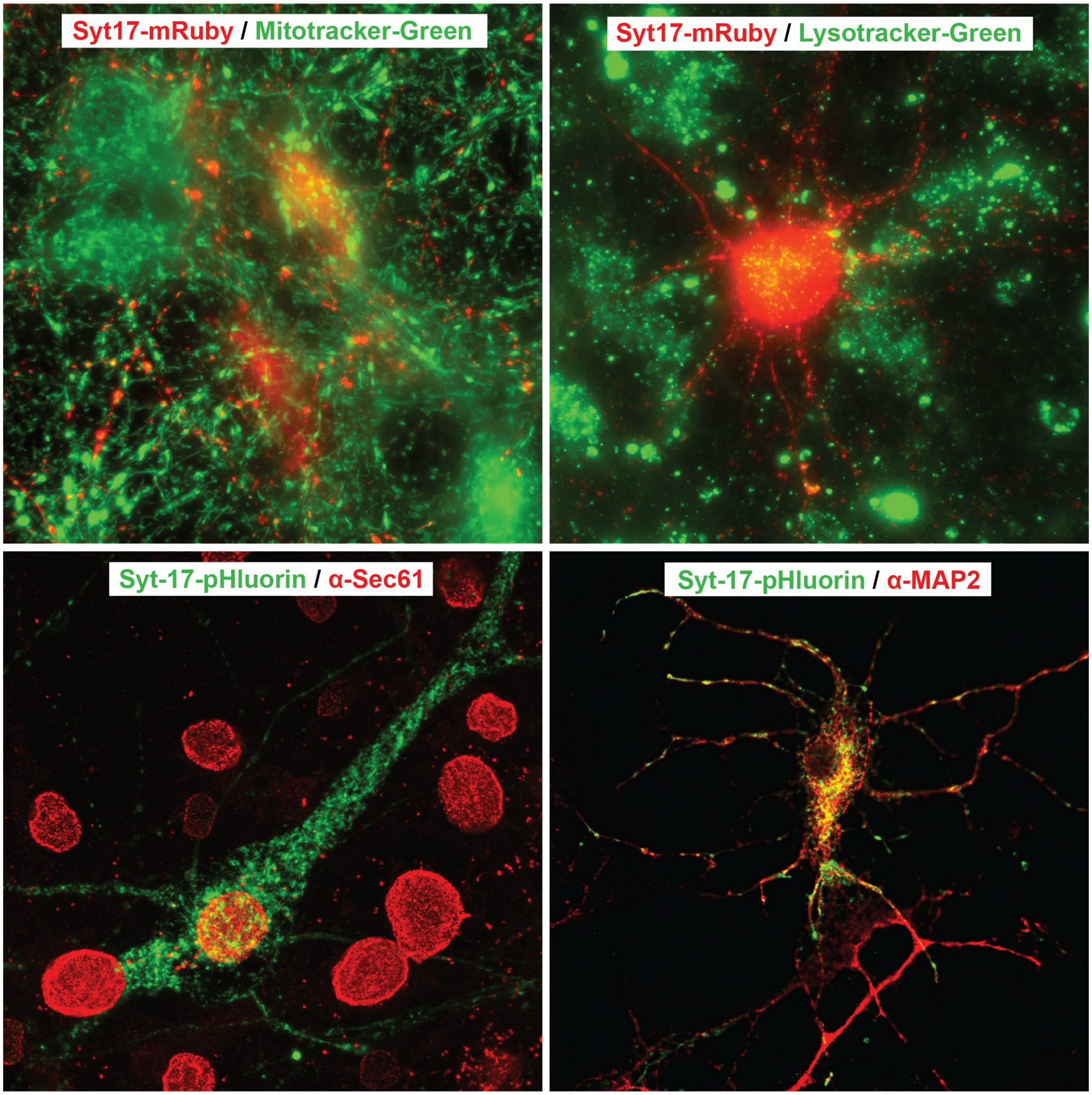

University of Wisconsin, Department of Neuroscience, WIMR2 1111 Highland Ave. (room 5505-mail; 9557-office), Madison, Wisconsin 53706
Tel. (608) 263-1762 • Fax. (608) 265-7821 • e-mail: chapman@wisc.edu
http://www.hhmi.org/research/investigators/chapman.html
http://neuro.wisc.edu/faculty/chapman.asp
http://www.neuro.wisc.edu/chapman/Chapman_Lab/Home.html

More generally, with respect to artificial off-targeting of tagged proteins at high levels of overexpression, this is an issue of which we are keenly aware. We should note that if mis-sorting of the tagged protein is occurring, it should become more evident over time, as additional copies of the protein accumulate. This is not the case for syt-17, we observed the same localization of the protein at 2-3DIV as at 14-15DIV. Additionally, if mis-sorting of the tagged protein is occurring, it should be more evident in the highest-expressing cells than the most weakly-expressing cells. Many of the transfections in our study employ the Ca^{2+}-phosphate method. This procedure results in some cells on a coverslip expressing the transfected construct more strongly than others, yet no change in the distribution of syt-17 across cells was observed.

Isoform-specific antibodies suitable for immunostaining do not exist for most members of the synaptotagmin family, and so for the time being the field necessarily relies on tagged proteins. To our knowledge, subcellular localization information of most synaptotagmin isoforms is based on overexpression approaches, including syt-5 (Dean et al., 2012), syt-6 (Wong et al., 2015), syt-9 (Xu et al., 2007), syt-10 (Cao et al., 2011), syt-11 (Wang et al., 2015), syt-14 (Doi et al., 2011), and syt-15 (Fukuda, 2003). Suffice to say, this is an open issue that we are all actively working on.

4. Fig3c: Expressing either of two mutants abolished the overexpression phenotype: these data are less informative as both mutant constructs are unlikely to have a dominant-negative effect. A new study showing failed rescue of the KO neuron phenotype with these mutants would support their conclusion.

We were not able to repeat these experiments due to low availability of knockout neurons. In any case, the conclusion we derive from this experiment does not depend on whether the experiment was performed in WT or KO neurons. The effect we observed with overexpression of syt-17 was an increase in axonal length, and the mutations we made to the protein were *loss-of-function* mutations. Expression of these mutants does not interfere with function of the wildtype protein, as axonal length is unaltered in neurons expressing the mutants. In other words, our data rule out the possibility of dominant-negative effects, because if these mutations were dominant-negative then their expression would produce axons *shorter* than control (GPF-only) neurons. This was not the case for any mutant (Fig. 3A-C). Had this experiment been conducted in KO neurons, one would not be able to distinguish "loss-of-function" from "dominant-negative" interpretations.

Finally, we are chiefly interested in the overexpression phenotype with respect to axon regeneration, and therefore conducting the experiment in WT neurons would thus appear more informative for future work.

University of Wisconsin, Department of Neuroscience, WIMR2 1111 Highland Ave. (room 5505-mail; 9557-office), Madison, Wisconsin 53706
Tel. (608) 263-1762 • Fax. (608) 265-7821 • e-mail: chapman@wisc.edu
http://www.hhmi.org/research/investigators/chapman.html
http://neuro.wisc.edu/faculty/chapman.asp
http://www.neuro.wisc.edu/chapman/Chapman_Lab/Home.html

5. Fig3e-3f: Enhanced regrowth capacity after injury is rather subtle. Even KO neurons display some degree of regeneration (FigS4b-c). Thus, rescue of KO neuron regeneration by expressing syt-17 is necessary.

Some residual growth in syt-17 KO neurons was expected, as all axons are sheared off during neuronal harvest and must regrow after plating. We did not mean to imply that syt-17 is necessary for all axonal regrowth, and have edited the manuscript text to clarify this point.

In the present manuscript, we demonstrated that the two main phenotypes of neurons lacking syt-17 (stunting of neurites, and augmentation of EPSCs) are fully recovered by expressing this protein (Figure 2C-D and Figure 7A-B, respectively). Respectfully, the logic prompting the proposed new rescue experiment, is not clear to us. We argue that conducting this experiment does not enhance our manuscript, as we have already demonstrated that:

- When neurons are overexpressing syt-17, axons regrow significantly more quickly than wildtype (Figure 3 D-F); this effect is statistically significant.
- When neurons are lacking syt-17, axons regrow significantly more slowly than wildtype (Supplementary Figure 4 B-C); this effect is statistically significant.

So, we have already shown that up- and downregulation of syt17 up- and downregulates axon regeneration. By repeating this experiment using KO neurons, the magnitude of the effect may be larger, but we argue that new insights will not be gained. In our opinion, demonstrating that an effect is bidirectional with respect to expression level is **more** informative than simply rescuing a loss of function.

There are other issues at play as well. This experiment has quite a high failure rate, as the low-volume microfluidic platform is exquisitely sensitive. Each animal yields sufficient neurons for six microfluidics, two to three of which typically grow. Of those that survive, typically a third of cultured microfluidics die once infected with virus. A further third typically die immediately after axotomy, meaning that overall, ~16% or less of seeded microfluidic devices potentially yield usable results. The data in Figure 3D-F, representing measurements from >600 axons, was a main focus of the responsible author for the better part of a year. We estimate it would take nearly a year to repeat the microfluidic experiments using KO neurons that do, and do not, over-express exogenous syt17. Also, repeating these experiments using KO neurons will require several dozen mice, and we feel that this loss of life is not clearly justified, again because we have already shown loss of syt17 decreases axon regrowth, and over-expression facilitates regrowth, so we are unable to see what new insights would be gained.

University of Wisconsin, Department of Neuroscience, WIMR2 1111 Highland Ave. (room 5505-mail; 9557-office), Madison, Wisconsin 53706
Tel. (608) 263-1762 • Fax. (608) 265-7821 • e-mail: chapman@wisc.edu
http://www.hhmi.org/research/investigators/chapman.html
http://neuro.wisc.edu/faculty/chapman.asp
http://www.neuro.wisc.edu/chapman/Chapman_Lab/Home.html

The referee notes that the regrowth acceleration effect itself was rather subtle. The effect (~24% increase in regrowth 48 hours post-axotomy) is indeed somewhat small, however we emphasize that this was expected, as other published genetic manipulations which augment axonal regrowth yield similar real-but-subtle effects. For example, overexpression of 14-4-4z produces a ~15% increase in regrown axon length (Kaplan et al., 2017), and overexpression of Rab11 produces a ~20-25% increase in regrown axon length (Koseki et al., 2017). It is fair to say that most individual "axon growth genes", including syt-17, have small effects on regrowth when modulated in isolation (ex., Blackmore, 2010; Sekine et al., 2018). At this stage, it is not possible to divine which combinations of small-effect genes will make effective therapeutic targets. Our data merely argue that syt-17 should be included on list of potential candidates.

6. FigS4d-e: While KO neurons display reduced growth in both axons and dendrites, the overexpression phenotype is specific to axons. This is an interesting observation, which might indicate a unique nature of syt-17-mediated secretary trafficking in axons vs dendrites. A high-resolution analysis of secretary cargos trafficking in axons vs dendrites might provide insights into this issue.

This is a very interesting point. To be perfectly honest, we originally hoped to use the UVR8-based probe employed in Figure 5 to quantify ER->Golgi trafficking separately in neuronal somata and dendrites. However, it immediately became apparent that the tools available to us were not able to robustly address this question. In the paper describing the probe (Chen et al., 2013), several images are shown using the probe to visualize secretory trafficking in dendrites (Fig. 3D of Chen et al., 2013). However, in our hands, only a small minority of cells exhibit substantial fluorescence in neurites (other than in the most proximal apical dendrite, where the Golgi would be contiguous with somatic Golgi), and what florescence can be observed photobleaches sufficiently quickly that extracting a stable time course is not possible. Despite investing significant time (and communicating with the corresponding author of Chen et al. directly for additional advice) we were not able to consistently extract dendritic time courses, and were forced to table that question. New tools – in which protein function can be rapidly perturbed in a neuron subcompartment-specific manner, and/or in which dendritic secretory trafficking can be targeted more specifically – will need to be engineered to answer the referee's question in a robust fashion.

(We should note that what we have written here should not be construed as "we failed to replicate Chen et al., 2013", a paper which we admire and rely upon in our own work.

That paper did not attempt to quantify continuous time courses from dendrites using the UVR8-based probe, and then subsequently compare those time courses across cells. This is a much more demanding application of the probe than that of Chen et al. [2013], which only showed still images in dendrites pre- and post-uncaging.)

7. In the experiments where multiple comparisons are made, t-tests were used to compute statistics rather than ANOVA tests with correction for multiple comparisons.

This has been corrected, thank you.

Reviewer #3:

1. From the data presented it is unclear if synaptotagmin-17 is indeed a synaptotagmin isoform. I think that the definition of a "synaptotagmin" is a protein with N-terminal transmembrane region and C-terminal tandem C2 domains. B/K protein, or Synaptotagmin-17, is lacking N-terminal transmembrane region. If the authors extended definition of synaptotagmins apply, then every Doc2 proteins should be called a synaptotagmin isoform. Therefore, in my opinion, B/K protein (Synaptotagmin-17) is not a synaptotagmin isoform and accordingly it displays unusual characteristics from those of synaptotagmins. This point needs further justification by the authors.

We thank the reviewer for raising this interesting conceptual point regarding the nomenclature of syt-17. The protein was originally termed as "B/K protein", on the basis of the presence of the mRNA in brain and kidney (Kwon et al., 1996). This term may or may not have been something of a misnomer, as the mRNA was later reported to be in prostate as well (Chin et al., 2006) – but in any case, regardless of the mRNA distribution, expression of the protein itself appears largely restricted to brain (Fukuda & Mikoshiba, 2001).

The central point here is that the modern categorization of this protein as a member of the synaptotagmin family was not a determination made by us, or by any other individual laboratory. The categorization of this protein as a member of the synaptotagmin family was a determination made independently by several large genomics consortiums, based on gene ontology and ancestry. Accordingly, all major bioinformatics databases, to our knowledge, describe the protein as synaptotagmin 17. These include, to choose a few examples:

- The HUGO Gene Nomenclature Committee https://www.genenames.org/data/gene-symbol-report/#!/hgnc_id/HGNC:24119

University of Wisconsin, Department of Neuroscience, WIMR2 1111 Highland Ave. (room 5505-mail; 9557-office), Madison, Wisconsin 53706
Tel. (608) 263-1762 • Fax. (608) 265-7821 • e-mail: chapman@wisc.edu
http://www.hhmi.org/research/investigators/chapman.html
http://neuro.wisc.edu/faculty/chapman.asp
http://www.neuro.wisc.edu/chapman/Chapman_Lab/Home.html

- Ensemble https://uswest.ensembl.org/Homo_sapiens/Gene/Summary?g=ENSG00000103528;r=16:19167971-19268334
- NCBI https://www.ncbi.nlm.nih.gov/nuccore/NM_016524
- Mouse Genome Informatics http://www.informatics.jax.org/marker/MGI:104966
- GeneCards https://www.genecards.org/cgi-bin/carddisp.pl?id_type=hgnc&id=24119

Given that most genes which have been the subject of mechanistic study possess more than one potential name or identifier, we believe that it is important for individual research groups to adhere to standardized, data-driven, gene nomenclatures whenever possible. Our use of the term "syt-17" is consistent with this philosophy, and further is consistent with other modern work that touched on this protein (ex., Sharma et al., 2015; Wong et al., 2015; Dean et al., 2012).

We note that most of these genomic databases include "B/K protein" as an alias, and/or cite older papers using the term "B/K protein". Additionally, in the introduction to our manuscript, we cite much of the seminal work that described this protein under the name "B/K protein", and we explicitly note this alternative name (paragraph three of the Introduction). Therefore, we do not think any researcher curious about the function of the protein will be unduly confused by the modern nomenclature.

2. I think knocking out B/K protein is very specific, but as B/K protein interacts with important Golgi and early endosome proteins, it is likely involved in the functions of the Golgi as well as endosomes. As the Golgi is essential for several cellular functions, impairing the Golgi function can produce many different types of physiological changes including those the manuscript described. These potential indirect effects of B/K or synaptotagmin-17 should be acknowledged.

We agree with this point, as secretory trafficking and endosomal recycling both have myriad functions in neurons. To the extent that we did not include sufficient caveats to this effect in our discussion section, we are remiss, and thank the reviewer for pointing this out. Accordingly, we have expanded the caveats in our text as follows:

> *However, we cannot formally rule-out the possibility that these changes in spine shape or other synaptic phenotypes are somehow secondary to dysfunction in the secretory pathway. Novel tools to selectively delete proteins from specific intracellular compartments are needed to fully disentangle these two possibilities, and confirm that the role of syt-17 in the phenotypes reported here is direct.*

University of Wisconsin, Department of Neuroscience, WIMR2 1111 Highland Ave. (room 5505-mail; 9557-office), Madison, Wisconsin 53706
Tel. (608) 263-1762 • Fax. (608) 265-7821 • e-mail: chapman@wisc.edu
http://www.hhmi.org/research/investigators/chapman.html
http://neuro.wisc.edu/faculty/chapman.asp
http://www.neuro.wisc.edu/chapman/Chapman_Lab/Home.html

FINAL COMMENTS:

Again, we thank all reviewers for their comments; their input greatly strengthened the manuscript. In the revised manuscript, changes to the text are highlighted in yellow. Additions and alterations have been made to Figure 3, Figure 7, Supplementary Figure 4 (only a small change to the coloring), and Supplementary Figure 8.

References:

Blackmore, M. G. *et al.* High content screening of cortical neurons identifies novel regulators of axon growth. *Mol. Cell. Neurosci.* **44**, 43–54 (2010).

Bowen, A. B., Bourke, A. M., Hiester, B. G., Hanus, C. & Kennedy, M. J. Golgi-independent secretory trafficking through recycling endosomes in neuronal dendrites and spines. *Elife* (2017). doi:10.7554/elife.27362

Brown, T. C., Correia, S. S., Petrok, C. N. & Esteban, J. A. Functional Compartmentalization of Endosomal Trafficking for the Synaptic Delivery of AMPA Receptors during Long-Term Potentiation. *J. Neurosci.* (2007). doi:10.1523/jneurosci.4258-07.2007

Brown, T. C., Tran, I. C., Backos, D. S. & Esteban, J. A. NMDA receptor-dependent activation of the small GTPase Rab5 drives the removal of synaptic AMPA receptors during hippocampal LTD. *Neuron* **45**, 81–94 (2005).

Buffa, L., Fuchs, E., Pietropaolo, M., Barr, F. & Solimena, M. ICA69 is a novel Rab2 effector regulating ER-Golgi trafficking in insulinoma cells. *Eur. J. Cell Biol.* **87**, 197–209 (2008).

Cao, P., Maximov, A. & Südhof, T. C. Activity-dependent IGF-1 exocytosis is controlled by the Ca(2+)-sensor synaptotagmin-10. *Cell* **145**, 300–11 (2011).

Chen, D., Gibson, E. S. & Kennedy, M. J. A light-triggered protein secretion system. *J. Cell Biol.* **201**, 631–640 (2013).

Chin, H. *et al.* Protein kinase A-dependent phosphorylation of B/K protein. *Exp. Mol. Med.* **38**, 144–152 (2006).

Dean, C. *et al.* Axonal and dendritic synaptotagmin isoforms revealed by a pHluorin-syt functional screen. *Mol. Biol. Cell* **23**, 1715–27 (2012).

Doi, H. *et al.* Exome sequencing reveals a homozygous SYT14 mutation in adult-onset, autosomal-recessive spinocerebellar ataxia with psychomotor retardation. *Am. J. Hum. Genet.* (2011). doi:10.1016/j.ajhg.2011.07.012

Fukuda, M. Molecular cloning and characterization of human, rat, and mouse synaptotagmin XV. *Biochem. Biophys. Res. Commun.* (2003). doi:10.1016/S0006-291X(03)00911-2

Fukuda, M. & Mikoshiba, K. The N-terminal cysteine cluster is essential for membrane targeting of B/K protein. *Biochem. J.* **360**, 441–8 (2001).

He, Z. & Jin, Y. Intrinsic Control of Axon Regeneration. *Neuron* (2016). doi:10.1016/j.neuron.2016.04.022

University of Wisconsin, Department of Neuroscience, WIMR2 1111 Highland Ave. (room 5505-mail; 9557-office), Madison, Wisconsin 53706
Tel. (608) 263-1762 • Fax. (608) 265-7821 • e-mail: chapman@wisc.edu
http://www.hhmi.org/research/investigators/chapman.html
http://neuro.wisc.edu/faculty/chapman.asp
http://www.neuro.wisc.edu/chapman/Chapman_Lab/Home.html

Hirling, H. Endosomal trafficking of AMPA-type glutamate receptors. *Neuroscience* **158**, 36–44 (2009).

Kaplan, A. *et al.* Small-Molecule Stabilization of 14-3-3 Protein-Protein Interactions Stimulates Axon Regeneration. *Neuron* (2017). doi:10.1016/j.neuron.2017.02.018

Koseki, H. *et al.* Selective rab11 transport and the intrinsic regenerative ability of CNS axons. *Elife* (2017). doi:10.7554/elife.26956

Kwon, O. J., Gainer, H., Wray, S. & Chin, H. M. Identification Of a Novel Protein Containing 2 C2 Domains Selectively Expressed In the Rat-Brain and Kidney. *FEBS Lett.* **378**, 135–139 (1996).

Sekine, Y. *et al.* Functional Genome-wide Screen Identifies Pathways Restricting Central Nervous System Axonal Regeneration. *Cell Rep.* (2018). doi:10.1016/j.celrep.2018.03.058

Sharma, K. *et al.* Cell type- and brain region-resolved mouse brain proteome. *Nat. Neurosci.* (2015). doi:10.1038/nn.4160

Wang, C. *et al.* Synaptotagmin-11 inhibits clathrin-mediated and bulk endocytosis. *EMBO Rep.* **17**, 47–63 (2016).

Wong, Y., Lee, C., Xie, W., Cui, B. & Poo, M. Activity-dependent BDNF release via endocytic pathways is regulated by synaptotagmin-6 and complexin. *Proc. Natl. Acad. Sci.* 4475–4484 (2015). doi:10.1073/pnas.1511830112

Xu, J., Mashimo, T. & Su, T. C. Synaptotagmin-1, -2, and -9: Ca2+ Sensor for Fast Release that Specify Distinct Presynaptic Properties in Subsets of Neurons. *Neuron* **54**, 567–581 (2007).

University of Wisconsin, Department of Neuroscience, WIMR2 1111 Highland Ave. (room 5505-mail; 9557-office), Madison, Wisconsin 53706
Tel. (608) 263-1762 • Fax. (608) 265-7821 • e-mail: chapman@wisc.edu
http://www.hhmi.org/research/investigators/chapman.html
http://neuro.wisc.edu/faculty/chapman.asp
http://www.neuro.wisc.edu/chapman/Chapman_Lab/Home.html

REVIEWERS' COMMENTS:

Reviewer #1 (Remarks to the Author):

The authors perfectly addressed all my points. From my point of view the manuscript can be published as it is.

Reviewer #2 (Remarks to the Author):

The revision (NCOMMS-19-00844) includes additional experimental data that addresses some of my concerns. I am sure the current work will facilitate future follow-up studies in advancing our mechanistic understanding of this important isoform of synaptotagmins in neurons.

Reviewer #3 (Remarks to the Author):

The authors have addressed the issues I raised. I don’t see any remaining issues that preclude publication
